# Genome analyses of colistin-resistant high-risk *bla*_NDM-5_ producing *Klebsiella pneumoniae* ST147 and *Pseudomonas aeruginosa* ST235 and ST357 in clinical settings

**DOI:** 10.1186/s12866-024-03306-4

**Published:** 2024-05-20

**Authors:** Absar Talat, Fatima Khan, Asad U. Khan

**Affiliations:** 1 Medical Microbiology and Molecular Biology Lab, Interdisciplinary Biotechnology Unit, Aligarh Muslim University, Aligarh, 202002 India; 2 Microbiology Department, JNMC and Hospital, Aligarh Muslim University, Aligarh, 202002 India

**Keywords:** Colistin, Antimicrobial resistance, *Klebsiella pneumoniae*, *Pseudomonas aeruginosa*, *bla*NDM, Antibiotic resistance

## Abstract

**Background:**

Colistin is a last-resort antibiotic used in extreme cases of multi-drug resistant (MDR) Gram-negative bacterial infections. Colistin resistance has increased in recent years and often goes undetected due to the inefficiency of predominantly used standard antibiotic susceptibility tests (AST). To address this challenge, we aimed to detect the prevalence of colistin resistance strains through both Vitek®2 and broth micro-dilution. We investigated 1748 blood, tracheal aspirate, and pleural fluid samples from the Intensive Care Unit (ICU), Neonatal Intensive Care Unit (NICU), and Tuberculosis and Respiratory Disease centre (TBRD) in an India hospital. Whole-genome sequencing (WGS) of extremely drug-resitant (XDR) and pan-drug resistant (PDR) strains revealed the resistance mechanisms through the Resistance Gene Identifier (RGI.v6.0.0) and Snippy.v4.6.0. Abricate.v1.0.1, PlasmidFinder.v2.1, MobileElementFinder.v1.0.3 etc. detected virulence factors, and mobile genetic elements associated to uncover the pathogenecity and the role of horizontal gene transfer (HGT).

**Results:**

This study reveals compelling insights into colistin resistance among global high-risk clinical isolates: *Klebsiella pneumoniae* ST147 (16/20), *Pseudomonas aeruginosa* ST235 (3/20), and ST357 (1/20). Vitek®2 found 6 colistin-resistant strains (minimum inhibitory concentrations, MIC = 4 μg/mL), while broth microdilution identified 48 (MIC = 32–128 μg/mL), adhering to CLSI guidelines. Despite the absence of mobile colistin resistance (*mcr*) genes, mechanisms underlying colistin resistance included *mgrB* deletion, phosphoethanolamine transferases *arnT*, *eptB*, *ompA*, and mutations in *pmrB* (T246A, R256G) and *eptA* (V50L, A135P, I138V, C27F) in *K. pneumoniae*. *P. aeruginosa* harbored phosphoethanolamine transferases *basS*/*pmrb*, *basR*, *arnA*, *cprR*, *cprS*, alongside *pmrB* (G362S), and *parS* (H398R) mutations. Both strains carried diverse clinically relevant antimicrobial resistance genes (ARGs), including plasmid-mediated *bla*_NDM-5_ (*K. pneumoniae* ST147) and chromosomally mediated *bla*_NDM-1_ (*P. aeruginosa* ST357).

**Conclusion:**

The global surge in MDR, XDR and PDR bacteria necessitates last-resort antibiotics such as colistin. However, escalating resistance, particularly to colistin, presents a critical challenge. Inefficient colistin resistance detection methods, including Vitek2, alongside limited surveillance resources, accentuate the need for improved strategies. Whole-genome sequencing revealed alarming colistin resistance among *K. pneumoniae* and *P. aeruginosa* in an Indian hospital. The identification of XDR and PDR strains underscores urgency for enhanced surveillance and infection control. SNP analysis elucidated resistance mechanisms, highlighting the complexity of combatting resistance.

**Supplementary Information:**

The online version contains supplementary material available at 10.1186/s12866-024-03306-4.

## Background

The antibiotic development pipeline has run dry, failing to compete with the constantly evolving antimicrobial resistance (AMR) crisis [1]. This ‘silent pandemic’ led to approximately 5 million mortalities in 2019 and is predicted to escalate drastically in 2050 with 10 million annual deaths [2]. Multidrug resistance (MDR) is a growing global public health concern, with multidrug-resistant bacterial pathogens emerging from various sources. This underscores the crucial need for judicious antibiotic use. Routine antimicrobial susceptibility testing is vital for identifying the appropriate antibiotics and screening for emerging MDR strains [3–8]. Global dissemination of MDR, hypervirulent, and high-risk clones poses a significant healthcare challenge, particularly in low and middle-income countries (LMICs) [2, 9]. Colistin, a crucial last-resort antibiotic for MDR gram-negative infections, has witnessed a surge in resistance over the past decade. The mechanisms of colistin resistance, though not fully elucidated, involve plasmid-mediated *mcr* genes, intrinsic factors, and genetic mutations [10, 11]. Since colistin’s bactericidal activity is associated with penetrating and disrupting the bacterial cell membrane, reduced colistin susceptibility is mediated by adding cationic molecules 4-deoxyaminoarabinose (LAra4N) and phosphoethanolamine (pEtN) to the lipopolysaccharide surface (LPS), particularly lipid A, extrusion of colistin through efflux pumps, and overexpression of outer membrane proteins. In *Klebsiella pneumoniae*, a transmembrane protein *mgrB* (47 amino acids) is a crucial factor intricately involved in the colistin resistance pathway. Insertional inactivation by interrupting IS element, truncation, or complete deletion of *mgrB* alters colistin susceptibility [12–16]. *mgrB* is the negative regulator of the two-component system *PhoPQ*. *PhoQ* is a sensor kinase activated by the low extracellular magnesium (Mg^2+^), acidic pH (5.5), or the presence of cationic antimicrobial peptides (AMPs). *PhoQ* activates *PhoP* through phosphorylation, which triggers the expression of the *pmrHFIJKLM* operon, also known as *pmrF*, *pbg,* or *arn* operon. The *pmrHFIJKLM* operon adds LAra4N to the lipid A of LPS. Several other factors contribute towards colistin resistance, notably, pEtN transferases such as *eptB* and *arnT*, alterations in two-component system *pmrAB*, *phoPQ*, and *crrAB*, *parRS*, *colRS*, upregulation of *acrA*, *tolC*, and *soxRS*, efflux pumps including *KpnEF*, *KpnG*, *acrAB* and sap protein systems, and porin ompA [12, 13, 15–20]. In *Pseudomonas aeruginosa,* LPS modifications reducing the surface anionic charge, the *arnBCADTEF-pmrE* (*pmrHFIJKLME*) encoding enzymes, which add LAra4N to lipid A, and alterations in *PmrAB*, *PhoPQ*, *ParRS*, and *CprRS* two-component regulatory systems are important mechanisms inducing colistin resistance [21].

The World Health Organisation (WHO) recognizes carbapenem-resistant *K. pneumoniae* and *P. aeruginosa* as ‘critical priority’ pathogens [22]. *K. pneumoniae* is a typical perpetrator of nosocomial, community-acquired, and opportunistic infections such as pneumonia, urinary tract infections (UTIs), bloodstream infections, and sepsis [23–26]. *Klebsiella pneumoniae*’s antimicrobial resistance primarily stems from: i. Enzyme production, notably β-lactamases like *bla*_NDM_, *bla*_KPC_, *bla*_OXA_. ii. Reduced cell permeability due to loss of outer membrane proteins (*OmpA*, *OmpK35*, *OmpK36*, *OmpK37*). iii. Overexpression of efflux pumps, expelling antibiotics from the cell. iv. Target modification, altering the antimicrobial agents’ targets [27]. β-lactamase emergence has significantly reduced the effectiveness of multiple antibiotics like carbapenems, cephems, clavams, monobactams, and penems, posing major public health challenges [28, 29]. The clonal type *K. pneumoniae* ST147 is a high-risk clone associated with MDR and pan-drug resistant chronic infections [9]. It was first reported in Hungary and Spain, genotypically characterized with fluoroquinolone resistance induced by *gyrA*S83I and *parC*S80I QRDR mutations and extended-spectrum β-lactamase gene *bla*_CTX-M-15_ [30, 31]. Later, it expanded globally, acquiring resistance against major antimicrobial classes, including ESBLs and carbapenems [9, 32, 33]. *K. pneumoniae* ST147 is endemic to India, Italy, Greece, and certain North African countries [9]. The high genomic plasticity, virulence, and extensive resistance acquisition make *K. pneumoniae* ST147 a potential epidemic trigger. *K. pneumoniae* ST147 carrying *bla*_NDM-1_ caused prolonged large outbreaks in Poland and the USA (2015–19) [34, 35]. Hypervirulent *K. pneumoniae* ST147 armed with a chimeric plasmid carrying resistance and virulence determinants caused an outbreak in Tuscany, Italy [36]. Beta-lactamase *bla*_NDM-5_ is endemic to India, and reportedly, *K. pneumoniae* ST147 acquired *bla*_NDM-5_ and *bla*_OXA-181_ in India, later disseminating it globally [37–39]. In India, colistin-resistant *K. pneumoniae* ST147 isolates have been reported in sepsis, acute kidney injury, and pulmonary tuberculosis [40–42].

*P. aeruginosa* is an urgent threat associated with severe nosocomial infections, bacteremia, sepsis, hospital-acquired pneumonia, and respiratory failure, especially in immunocompromised patients, diabetic ulcers, burn wounds, corneal ulcers, and surgical wounds [43]. It colonizes the lungs of cystic fibrosis (CF) patients, leading to pulmonary failure [44]. In *Pseudomonas aeruginosa*, antibiotic resistance is driven by three main mechanisms: intrinsic, acquired, and adaptive. i. Intrinsic resistance is due to reduced outer membrane permeability and presence of antibiotic efflux pumps. ii. Acquired resistance arises from horizontal gene transfer of antibiotic resistance genes (ARGs), mutations leading to increased expression of efflux pumps and β-lactamases, and alterations in antibiotic target sites. Iii. Adaptive resistance, which is transient and induced by external factors like stress or specific antibiotics, involves regulatory pathways, changes in gene expression, protein production, or modifications to antibiotic target [45]. *P. aeruginosa* ST235 and ST357 are widespread high-risk clones, with ST235 being the most prevalent. Their capability to acquire resistance against major antibiotics, including beta-lactamases, carbapenemases, and aminoglycosides, is a significant factor associated with high morbidity and mortality. The production of *ExoU* cytotoxin is a major virulence factor contributing to high lethal potential [46, 47].

Virulence factors (V.F.s) are crucial for enhancing bacterial infectivity by promoting strong attachment and colonization within host cells, disrupting tissue integration, evading the host immune response, and exploiting nutrient availability [48]. Classical and Hypervirulent *Klebsiella pneumoniae* (HVKP) strains differ in virulence, identified through a string test and gene-carrying plasmids. Key V.F.s include fimbriae adhesion, capsule, lipopolysaccharide (LPS), and siderophores, aiding the attachment, evasion from immunity, enhanced survival and colonization [27].

In *P. aeruginosa*, virulence factors (V.F.s) are categorized into three main categories: i. Bacterial surface structures including pili, flagella, secretion systems (Types 1, 2, 3, 5, and 6), and outer membrane proteins (e.g., lipopolysaccharide). These aid in attachment, motility, immune evasion, toxin delivery, and biofilm formation. ii. Secreted factors such as exopolysaccharides (Alginate, *Pel*, *Psl*), siderophores (Pyoverdine, Pyochelin), proteases (*AprA*, *LasA*, *LasB*, Protease IV), and toxins (*ExoS*, *ExoT*, *ExoU*, *ExoY*). These contribute to adhesion, immune evasion, growth promotion, and virulence induction. Iii. Virulence factor-mediated interactions between bacterial cells regulate quorum sensing and biofilm production, aiding in evasion of host immune responses [49].

Biofilms, intricate three-dimensional structures formed by *P. aeruginosa* and *K. pneumoniae*, enhance their pathogenicity and resistance to antimicrobial agents. The extracellular matrix, comprising polysaccharides, proteins, nucleic acids, lipids, and extracellular DNA (eDNA), supports bacterial growth within biofilms. In *P. aeruginosa*, virulence factors like alginate, *Psl*, and *Pel* aid in biofilm formation [49, 50]. Similarly, in *K. pneumoniae*, biofilm formation is influenced by factors such as the capsule, type 1 and type 3 fimbriae, and the *Escherichia coli* common pilus (ECP) fimbriae gene cluster [51]. Quorum sensing mechanisms regulate biofilm formation by controlling the synthesis of fimbriae, exopolysaccharides, and adhesins through signaling molecules, profoundly impacting both inter- and intra-species communication [52, 53].

Indian hospitals are one of the largest consumers of polymyxins, driven by the increasing cases of carbapenem-resistant *K. pneumoniae*, *P. aeruginosa*, and *Acinetobacter baumannii* infections [54]. Colistin resistance often goes unnoticed and is underreported in hospitals in India due to the incompetence of predominantly used standard antibiotic susceptibility tests such as Vitek and disk diffusion [55]. In this comprehensive whole-genome study, we investigated clinical isolates sourced from blood cultures of sepsis patients and pleural fluid, as well as tracheal aspirate samples from tuberculosis patients, collected during 2020–21. Our primary objective was to elucidate the factors contributing to colistin resistance within an Indian hospital setting, focusing on predominant bacterial species and the prevalence of other antibiotic resistance determinants. Through thorough analysis, we aimed to identify mechanisms of colistin resistance, including horizontal gene transfer facilitated by plasmid-mediated antibiotic resistance genes (ARGs), insertion sequence (IS) elements, virulence determinants, genomic islands, and bacteriophages. Additionally, we investigated the presence of CRISPR elements to assess bacterial immune responses against foreign genetic materials. Furthermore, we employed pangenome analysis to characterize the phylogenetic diversity of the bacterial isolates under study. Moreover, we sought to observe the differences between colistin resistance detection using Vitek2, which has previously yielded false negatives, and antimicrobial susceptibility testing (AST) through broth micro-dilution, aiming to provide a more accurate assessment of colistin resistance in our sample population.

## Methods

### Wet lab section

#### Isolation and antimicrobial susceptibility tests (AST) through VITEK2 and broth micro-dilution

The blood, pleural fluid, and tracheal aspirate (1748 samples) were inoculated on Luria Bertani agar (Hi Media, India) and incubated for 18 h at 37 °C. The pure colonies were picked by a sterile loop, inoculated in 5 mL LB broth and incubated on a shaker at 37 °C for 18 h. 0.3 mL of overnight culture was mixed with 0.7 mL of glycerol in sterile vials and stored at − 80 °C for future use. Gram-staining was performed to detect gram-positive and gram-negative isolates [49, 56]. The preliminary identification and antimicrobial susceptibility test against colistin and other antimicrobials (amikacin, amoxicillin, ampicillin, cefepime, cefoperazone/sulbactam, ceftazidime, ceftriaxone, ciprofloxacin, colistin, gentamicin, imipenem, levofloxacin, meropenem, piperacillin/tazobactam, ticarcillin/clavulanic acid+ cefotaxime, tigecycline, and trimethoprim/sulfamethoxazole) on isolates was performed using a semi-automated commercial system VITEK2 Advanced Expert system (BioMérieux, Marcy l’Étoile, France), using the European Committee on Antimicrobial Susceptibility Testing (EUCAST) breakpoints (http://www.eucast.org/clinical_breakpoints) [57]. The minimum inhibitory concentration (MIC) of colistin (Hi Media, India) and other antimicrobials including amikacin (Hi Media, India), aztreonam (Sigma-Aldrich, USA), cefazolin (Hi Media, India), cefepime (Hi Media, India), cefoxitin (Sigma-Aldrich, USA), ceftazidime (Sigma-Aldrich, USA), ceftriaxone (Sigma-Aldrich, USA), ciprofloxacin (Sigma-Aldrich, USA), colistin (Hi Media, India), Fosfomycin (Hi Media, India), gentamicin (Hi Media, India), imipenem (Hi Media, India), levofloxacin (Hi Media, India), meropenem (Hi Media, India), piperacillin (Hi Media, India), tetracycline (Hi Media, India), and ticarcillin-clavulanate (Hi Media, India) against the isolates was determined by broth microdilution as well, using cation-adjusted Mueller-Hinton broth (MHCB) [55, 58] (Additional Table 1). The results were interpreted according to the Clinical and Laboratory Standards Institute (CLSI) guidelines [59]. The classification of isolates into multi-drug resistant (MDR), extensively drug-resistant (XDR) and pandrug-resistant (PDR) was done according to Magiorakos et al. [60]. The MAR (Multiple Antibiotic Resistance) index was computed and analyzed following the methodology outlined by Krumperman, employing the formula: a/b. Here, ‘a’ denotes the count of antibiotics for which an isolate exhibited resistance, while ‘b’ stands for the total number of antibiotics subjected to testing [61]. The whole-cell DNA from the strains was obtained by collecting pure colonies from a culture plate and suspending them in 100 μL of nuclease-free water. Subsequently, the mixture was heated at 95 °C for 15 minutes in a thermal block and then centrifuged at 10,000 rpm at 4 °C for 10 minutes [62]. Gene amplification was performed using polymerase chain reaction (PCR) with gene-specific primers targeting *mcr*-1. The primers used were *mcr*-F(forward) 5′-CGG TCA GTC CGT TTG TTC-3′ and *mcr*-R (reverse) 5′-CTT GGT CGG TCT GTA GGG-3′ [63]. The PCR products were mixed with gel loading dye and run on 1% agarose gels in 1x TAE buffer. Ethidium bromide was added for visualization, and gel images were captured using a gel documentation system (Gel Doc) .

### Dry lab section

#### DNA extraction, library preparation, sequencing, and quality check

The DNA of colistin-resistant isolates was extracted using a QIAGEN DNeasy PowerSoil kit (Qiagen, Germany) according to the manufacturer’s protocol and subjected to a Nano Drop™ 2000 Spectrophotometer (ThermoFisher Scientific, USA) for checking RNA and protein contaminants [64]. The standard set for quality DNA is approximately 1.8 to 2 at 260/280 nm. QUBIT 3.0 Fluorometer (ThermoFisher Scientific, USA) quantified the extracted DNA using dS DNA HS (High Sensitivity) Dye. One hundred nanograms of intact DNA was enzymatically fragmented using Covaris, targeting 200–300 bp fragment size. The DNA fragments undergo end repair, where the mix converts the overhangs resulting from fragmentation into blunt ends. The end-repair mix’s 3′ to 5′ exonuclease activity removes the 3′ overhangs, and polymerase activity fills in the 5′ overhangs. The blunt-ended fragments are adenylated, adding a single ‘A’ nucleotide to the 3′ ends. The adenylated fragments loop adapters were ligated and cleaved with a uracil-specific excision reagent (USER) enzyme. The AMPure beads (Beckman Coulter Life Sciences, USA) further purified the DNA. The polymerase chain reaction (PCR) with 6 cycles using NEBNext Ultra II Q5 master mix (New England BioLabs®Inc., USA), Illumina universal primer (Illumina, USA), and sample-specific octamer primers amplified the DNA. The AMPure beads cleaned the amplified DNA, and the final DNA library was eluted in 15 μL of 0.1X Tris EDTA buffer. QUBIT 3.0 Fluorometer quantified 1ul of the library using dS DNA HS reagent [65]. 1 μL of the library was loaded on an Agilent DNA 7500 chip, and fragments were analyzed by Agilent 2100 Bioanalyzer. Illumina HiSeq 4000 (Illumina, USA) sequenced the DNA with a 2 × 150 bp paired-end run.

FASTQC v0.11.9 and MultiQC v1.10.1 checked the quality of raw reads [66, 67]. Trimgalore v0.6.6 removed the adapter and other contaminations (https://github.com/FelixKrueger/TrimGalore). The integration of both dry lab (in silico) and wet lab (in vitro) studies, utilizing a variety of databases, software, and tools (as illustrated in the following flowchart) enhance accuracy and minimize biases [68].


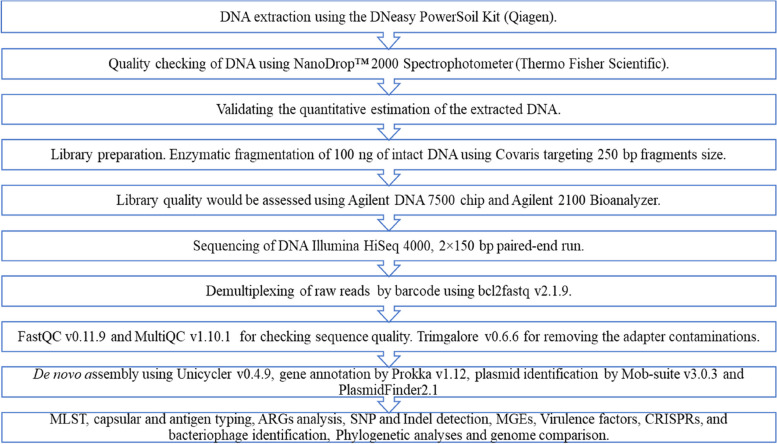


### Assembly and annotation of raw reads

The raw reads were assembled de novo (primary assembly) using Unicycler v0.4.9 [69]. The identity of isolate was further confirmed using 16S rRNA, and the average nucleotide identity (ANI) which was calculated from pair-wise comparisons of all sequences shared between the query and reference strain [70, 71]. The PubMLST web server (https://pubmlst.org/bigsdb?db=pubmlst_rmlst_seqdef_kiosk) identified the bacterial isolates [72]. Contiguator assembled the raw reads by mapping the contigs over a reference genome (secondary assembly) retrieved from the NCBI database, NZ_CP042858.1 for *K. pneumoniae*, NZ_CP053706.1, and NZ_LN831024.1 for *P. aeruginosa* [73]. PROKKA (Prokaryotic Genome Annotation) v1.12 annotated all the genomic features of the de novo assembled genome [74]. Mob-suite v3.0.3 distinguished and partitioned plasmid and nuclear sequence [75]. Plasmid types were further identified using PlasmidFinder2.1 (https://cge.food.dtu.dk/services/PlasmidFinder/) [76, 77].

### MLST, capsular, and antigen typing

The multi-locus sequence typing of *K. pneumoniae* for seven housekeeping genes *rpoB*, *gapA*, *mdh*, *pgi*, *phoE*, *infB*, and *tonB,* and *P. aeruginosa* for *acsA*, *aroE*, *guaA*, *mutL*, *nuoD*, *ppsA* and *trpE* was performed using the command-line version of mlst (https://github.com/tseemann/mlst) against PubMLST typing schemes based on Bacterial Isolate Genome Sequence database (BIGSdb) [72]. Kleborate screened the *K. pneumoniae* strains and predicted the K (capsule) and O antigen (LPS) serotypes [78]. The *Pseudomonas aeruginosa* serotyper (PAst) v1.0 (https://cge.food.dtu.dk/services/PAst/) identified the serogroup of *P. aeruginosa* by running a BLAST search on the O-specific antigen (OSA) gene cluster [77, 79].

### SNP and Indel detection

Snippy v4.6.0 (https://github.com/tseemann/snippy) identified the SNPs and indels conferring colistin resistance by mapping against colistin-susceptible reference whole genomes extracted from the NCBI database (https://www.ncbi.nlm.nih.gov/), NC_009648.1 *K. pneumoniae* subsp. *pneumoniae* MGH 78578 (ATCC® 700721™) and NZ_CP017149.1 *P. aeruginosa* strain (ATCC® 15692™). The SNPs rendering deleterious amino acid changes were assigned by SIFT (Sorting Intolerant From Tolerant) [80].

### ARGs identification

Resistance Gene Identifier (RGI) v6.0.0 identified the antibiotic resistance genes with 80% similarity and 90% identity in the isolates against using the Comprehensive Antibiotic Resistance Database (CARD) [81].

### Plasmids and IS elements

PlasmidFinder v2.1(https://cge.food.dtu.dk/services/PlasmidFinder/) identified the plasmids and mobile genetic elements were identified by Mobile Element Finder v1.0.3 (https://cge.food.dtu.dk/services/MobileElementFinder/).

### Virulence factors and pathogenicity toward humans

Virulence factors were identified by ABRicate v1.0.1 (https://github.com/tseemann/abricate) using the Virulence Factor Database (VFDB) with a cut-off coverage of 80 and 70% identification. The pathogenicity towards humans was predicted by PathogenFinder v1.1 (https://cge.food.dtu.dk/services/PathogenFinder/).

### CRISPRs, bacteriophages, and genomic islands interspersed in the bacterial genome

CRISPR (Clustered regularly interspaced short palindromic repeat) arrays and Cas proteins were identified by CRISPRCasFinder (https://crisprcas.i2bc.paris-saclay.fr/CrisprCasFinder/Index) [82]. Phigaro v2.2.6 detected and annotated prophage sequences within the bacterial genome. Genomic islands were individually identified using IslandViewer 4 (https://www.pathogenomics.sfu.ca/islandviewer/) and compared using IslandCompare (https://islandcompare.ca/).

### Phylogenetic analyses

Phylogenetic relationship was obtained using Roary v3.13.0 (https://github.com/sanger-pathogens/Roary) with a blastP identity cut-off criterion of 90%. The genes present in more than 99% of the isolates were assigned as core genes. Gubbins v 3.3 removed the recombination events (https://github.com/nickjcroucher/gubbins).

### Comparative genome analyses

The genome of *K. pneumoniae* strains and *P. aeruginosa* strains were compared against NC_009648.1 *K. pneumoniae* subsp. *pneumoniae* MGH 78578 (ATCC® 700,721™) and NZ_CP017149.1 *P. aeruginosa* strain (ATCC® 15,692™) respectively using Proksee (https://proksee.ca/).

The figures were generated in R using cowplot, ggplot2, pheatmap and Easyfig v.2.1 (https://mjsull.github.io/Easyfig/).

### Statistical analyses

The chi-square test was conducted to analyze the results using R v.4.1.2. The significance level was *p* < 0.05.

## Results

The primary objective of our study was to explore colistin resistance in XDR and PDR clinical isolates obtained from sepsis and tuberculosis patients in an Indian hospital. Notable observations include the discordance in colistin resistance detection between Vitek®2 and broth microdilution methods. Whole-genome sequencing unveiled a substantial prevalence of colistin-resistant strains, with 17 demonstrating extensive drug resistance and 3 displaying pan-drug resistance. Detailed analyses encompassing genomic features, plasmid sequences, molecular resistance determinants, virulence factors distribution, and CRISPR/Cas systems shed light on the underlying mechanisms of colistin resistance in *K. pneumoniae* and *P. aeruginosa* strains.

### Phenotypic characteristics of the recovered isolates

#### Clinical data and phenotypic characterisation of colistin-resistant bacterial isolates

We investigated colistin resistance in the isolates retrieved from blood culture of sepsis patients, pleural fluid, and tracheal aspirate culture of patients suffering from tuberculosis in Jawaharlal Nehru Medical College and Hospital (J.N.M.CH.), Aligarh, Uttar Pradesh, India, during the year 2020–2021. During this period, 1784 blood, tracheal aspirate, and pleural fluid samples were collected from Tuberculosis and Respiratory Disease centre (TBRD), Intensive Care Unit (ICU), and Neonatal Intensive Care Unit (NICU) for antibiotic susceptibility tests (AST), out of which 214 samples showed positive growth. AST through automated Vitek®2, detected only six colistin-resistant strains (MIC = 4 μg/mL). AST, through the broth microdilution method according to the latest CLSI guidelines, demonstrated 48 strains as colistin-resistant, out of which 17 strains were identified as extremely drug-resistant (XDR) and three as pan-drug resistant (PDR) [60]. Colistin resistance was high for these 20 strains, with a MIC range between 32 and 128 μg/mL (Additional Table 1). According to MAR index, *K. pneumoniae* ST147 isolates (MAR index = 0.846) were resistant to approximately 84.6% of the antibiotics included in the study. *P. aeruginosa* ST235 (MAR index = 1.0) exhibited resistance against all the tested antibiotics whereas *P. aeruginosa* ST357 (MAR index = 0.923) displayed resistance against approximately 92.3% of the tested antibiotics [61]. PCR amplification for detecting *mcr*-1 was negative for all the isolates.

### Genomic features identical in colistin-resistant strains

The whole genome of 20 XDR strains was sequenced for analysis. The GC content was comparable across all the strains in each ST lineage (Additional Table 2). All the sequenced strains were identified as AMR-high-risk clones, 16 as *K. pneumoniae* ST 147 (Additional Table 3), three as *P. aeruginosa* ST235 (AK-624, 625, 628), and one as *P. aeruginosa* ST357 (AK-631). All the strains were predicted as human pathogens. The *K. pneumoniae* ST147 belongs to clonal group CG147, which has disseminated globally, causing several outbreaks in recent years. The identified capsular types were KL10, wzi420, and O3/O3a O locus in all the *K. pneumoniae* strains*.* None of the strains possessed hypervirulence genes *rmpA* and *rmpA2. P. aeruginosa* ST235 is the founder of the CC235 clonal complex, and *P. aeruginosa* ST357 is the founder of the CC357 clonal complex. All the *P. aeruginosa* strains displayed O11 serogroup (Additional Table 3).

#### Identification of nuclear and plasmid sequences

Eight plasmids were reconstructed in *K. pneumoniae* strains, further identified as IncFIB, IncFIIA, IncR, IncFIA, and ColRNAI. In addition to the plasmids mentioned above, PlasmidFinder-2.1 identified Col(phAD28) in AK-618, 620, 626, and 630 (Additional Table 4). In *P. aeruginosa* strains (AK-624, 625, 628 and 631), the reconstructed plasmid was identified as IncP by Mob-suite v3.0.3. PlasmidFinder-2.1 didn’t detect any plasmid in *P. aeruginosa* strains (Additional Table 4).

#### Molecular determinants of colistin resistance indicate *mgrB* deletion and mutations in chromosomally mediated factors

**Fig. 1:**
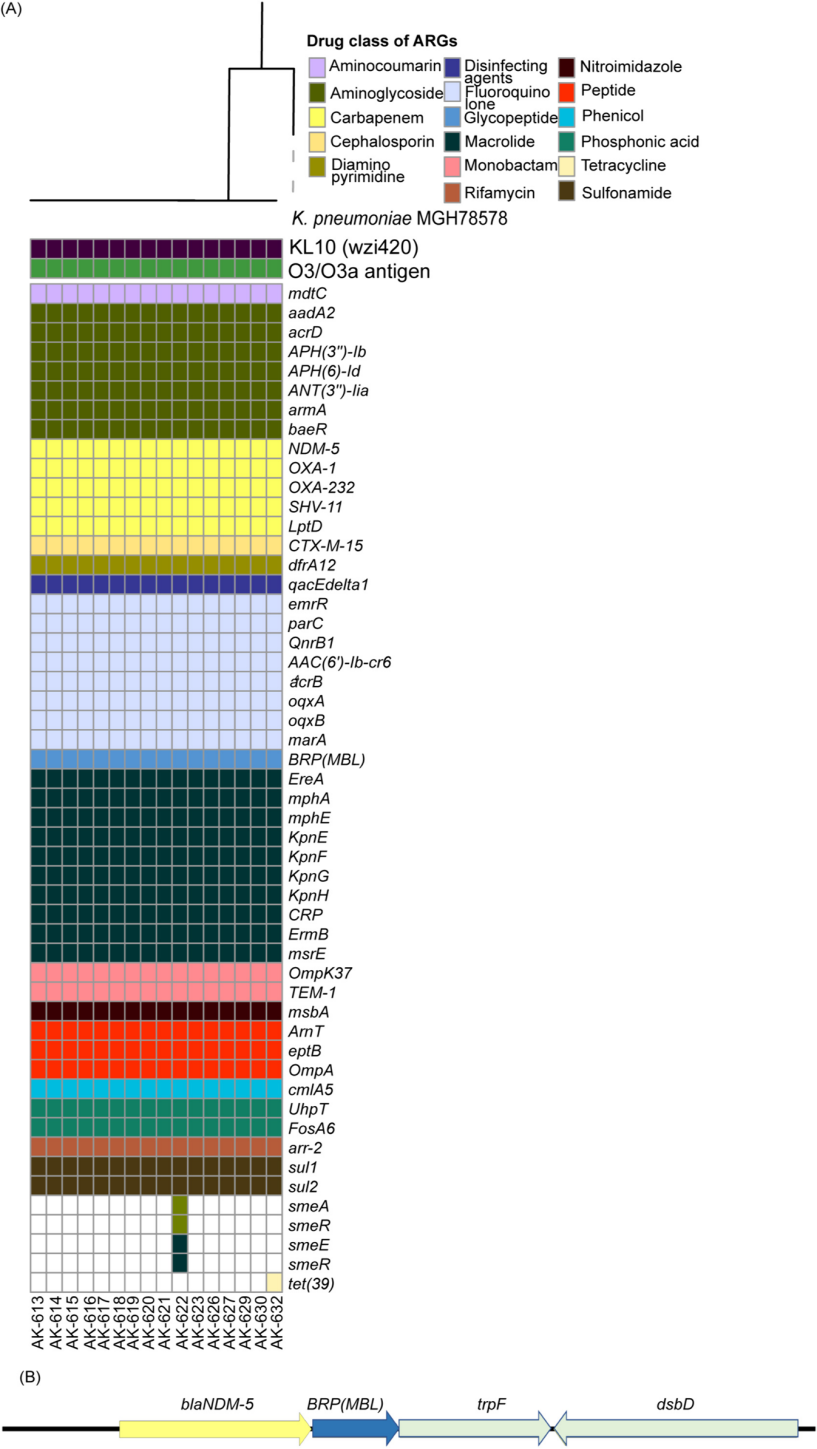
**A** Antibiotic resistance genes in *K. pneumoniae* strains. The phylogenetic relatedness among the strains was determined by recombination free alignment of single-nucleotide variants maximum-likelihood phylogenetic tree rooted at *K. pneumoniae* MGH 78578 (ATCC® 700,721™). All strains possessed KL10 (*wzi*420) capsular type and O3/O3a antigen. **B** Genetic characterization of *bla*_NDM-5_. Bleomycin resistance gene BRP(MBL), Phosphoribosylanthranilate (PRA) isomerase (TrpF) and disulfide isomerase-like protein (DsbD) were flanked downstream of *bla*_NDM-5_

Although no *mcr* genes were detected, several factors inducing colistin resistance were identified in *K. pneumoniae* and *P. aeruginosa* strains. The *pmr* phosphoethanolamine transferase encoding genes *arnT*, *eptB, ompA* bacterial porin with reduced permeability to peptide antibiotics were detected in *K. pneumoniae* (Fig. 1A). None of the *K. pneumoniae* strains possessed *mgrB*, indicating deletion. The SNP/indel detection against susceptible strain confirmed the complete deletion of *mgrB* due to bidirectional gene fusion.

There were no mutations detected in the *PhoPQ* two-component system. Mutations at two amino acid positions in *pmrB* (T246A and R256G) and four amino acids in *eptA* (V50L, A135P, I138V, and C27F) were found in all the *K. pneumoniae* isolates. Missense mutations in *arnA*, *arnB*, *arnT*, and *kpnF* were also detected (Additional Table 5A). Frameshift mutations in ompK35 increase the carbapenem resistance [83].

**Fig. 2:**
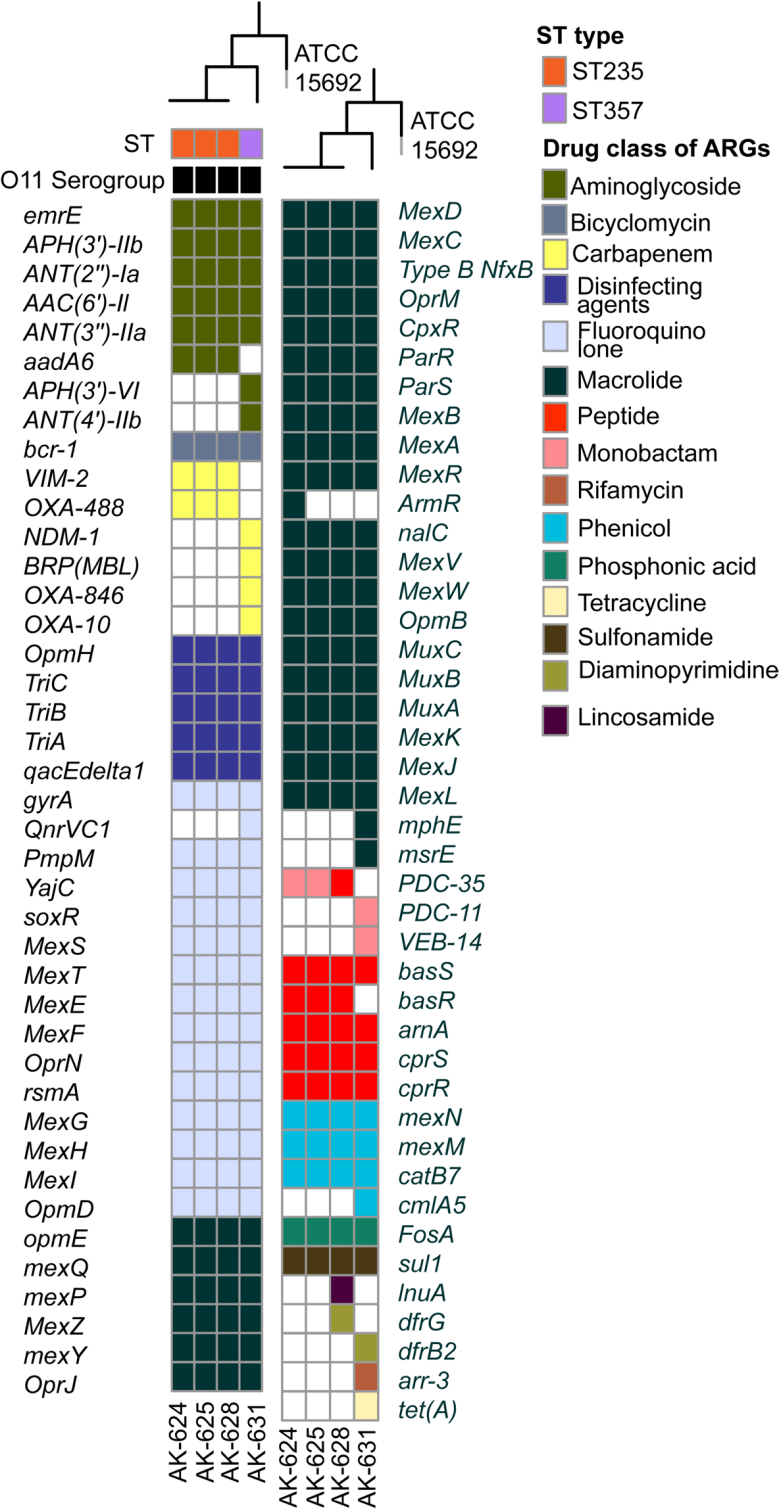
**A** Antibiotic resistance genes in *P. aeruginosa* strains. The phylogenetic relatedness among the strains was determined by recombination free alignment of single-nucleotide variants maximum-likelihood phylogenetic tree rooted at *P. aeruginosa* strain (ATCC® 15,692™). Three strains were ST235 and one was ST357. All strains possessed O11 serogroup. **B** Genetic characterization of *bla*_NDM-1_. No adjacent genes were identified in the contig carrying *bla*_NDM-1,_ owing to limited sequencing depth

Five *pmr* phosphoethanolamine transferase genes *basS* (also known as *pmrB*), *basR*, *arnA*, *cprR,* and *cprS* were identified in *P. aeruginosa* strains (Fig. 2A). These genes modulate the addition of phosphoethanolamine to lipid A, creating a cationic charge on the cell membrane inhibiting the binding of positively charged colistin. Mutations in *arnA*, *arnB*, *arnC*, *arnD*, *arnF*, *arnT*, *pmrA*, *pmrB,* and *parS* were identified in *P. aeruginosa* (Additional Table 5B and C).

## Distribution of virulence factors and antimicrobial resistance genes (ARGs) among the recovered isolates

### Prevalence of ARGs against all the major antibiotic classes

All the strains were detected as extended-spectrum-lactamase (ESBL)- and carbapenem-resistant strains. The resistance profile and total number of ARGs in all the *K. pneumoniae* strains were comparatively similar, conferring resistance against approximately all the major classes of antibiotics, including aminocoumarin, aminoglycoside, carbapenem, cephalosporin, macrolide, monobactam, peptide and sulfonamide (Fig. 1A and Additional Table 7). The predominant resistance mechanisms were antibiotic efflux and antibiotic inactivation. All the *K. pneumoniae* strains carried metallo-β-lactamase *bla*_NDM-5_, extended-spectrum β-lactamases *bla*_SHV-11_, *bla*_CTX-M-15_ and carbapenemases *bla*_OXA-1_ and *bla*_OXA-232_. The *bla*_NDM-5_ varies from *bla*_NDM-1_ at two positions, i.e., V88L and M154L, and is endemic to India [37, 39]. *bla*_NDM-5_ was mediated by the IncFII plasmid (Fig. 1B). *K. pneumoniae* ST147 is often characterized by *gyrA* S831 and *parC* S80I QRDR mutations conferring quinolone resistance and *bla*_CTX-M-15_ mediated by ISEcp1. *K. pneumoniae* strains in this study possessed *gyrA* D87A and *parC* with S80I and N304S mutation. *bla*_CTX-M-15_ was mediated by the IncR plasmid.

The resistome of *P. aeruginosa* strains was distinct from that of *K. pneumoniae*. The total number of ARGs was comparatively higher (Fig. 2A). In *P. aeruginosa* ST235, we detected metallo-β-lactamase *bla*_VIM-2_ and carbapenemase *bla*_OXA-488_. In *P. aeruginosa* ST357, *bla*_NDM-1_, *bla*_OXA-10_ and *bla*_OXA-846_ were identified. *bla*_NDM-1_ was chromosomally mediated, inferred through its presence in the nuclear partition of the genome, and further confirmed with PlasmidFinder2.1 (Fig. 2B).

### Virulence factors (V.F.S)

All *K. pneumoniae* strains possessed 19 virulence factors, notably, *entB* and *entA* encoding siderophores system enterobactin, *ompA*, pilus chaperones encoding genes *yagZ/ecpA*, *yagY/ecpB*, *yagV/ecpE* and *yagX/ecpC*, polymerized tip adhesin *yagW/ecpD*, iron-enterobactin ABC transporter permease *fepG,* and ferrienterobactin ABC transporter ATPase *fepC* (Additional Table 6).

*P. aeruginosa* AK-624, AK-625, and AK-628 had an extensive array of 224 virulence factors contributing to pathogenesis (Additional Table 6). AK-631 carried 216 virulence factors. Alginate biosynthesis proteins encoding genes (*alg44*, *alg8*, *algA*, *algB*, *algC*, *algD*, *algE*, *algF*, *algG*, *algI*, *algJ*, *algK*, *algL*, *algP/algR3*, *algQ*, *algR*, *algU*, *algW*, *algX*, *algZ*), type III, IV and VI secretion system associated virulence factors were prevalent. Exoenzymes Exo T, ExoU, and ExoY of the type III secretion system were present. ExoU is a significant virulence factor causing acute epithelial injury, rapid cell lysis, and destruction of the host cell plasma membrane [46].

### IS elements associated with ARGs

IS5075, ISEc29, ISKpn1, and ISKpn14 were common in all the ColRKp (colistin-resistant *K. pneumoniae*) strains (Additional Table 4). The beta-lactamase *bla*_TEM-1_, aminoglycoside resistance genes *aph(6)-Id* and *aph(3″)-Ib*, and sulfonamide resistance gene *sul2* were present adjacent to each other, flanked by IS5075. Aminoglycoside resistance gene *armA* and macrolide resistance genes *msrE* and *mphE* were flanked by ISEc29. In *P. aeruginosa*, ISPa6, ISPa86, IS6100 and ISpa7 were identified. In AK-631, the phosphoethanolamine transferases *cprR* and *cprS* were flanked by ISPre2, aiding in the HGT of these genes conferring colistin resistance. Only the *PmpM* gene encoding for fluoroquinolone was flanked by ISPa86. Tn6196 was associated with *aph(3′)-VI*, ISPa6 with *pmpM*, phosphonic acid *fosA*, and phenicol resistance gene *mexM* and *mexN*, and IS26 with fluoroquinolone resistance gene *qnrVC1*.

### CRISPR/Cas systems

All the *K. pneumoniae* strains were accompanied by CRISPR/Cas Class I with 43 spacers. Class I CRISPR with 9, 17, and 7 spacers were detected in *P. aeruginosa* ST357 (Additional Table 3).

### Genomic Islands (GIs)

**Fig. 3:**
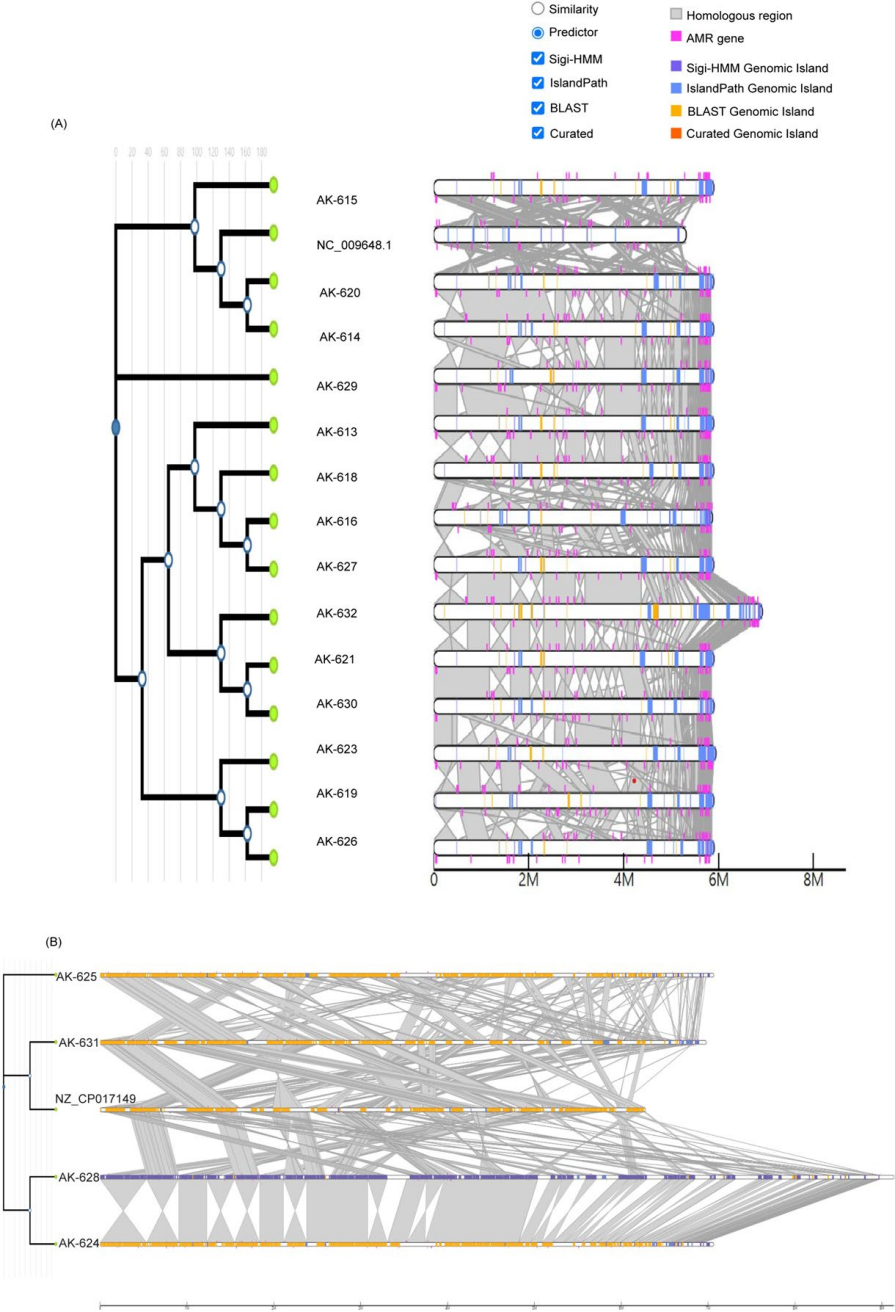
**A** Genomic islands in *K. pneumoniae* strains. GIs screened against *K. pneumoniae* subsp. *pneumoniae* MGH 78578 predicted common genomic islands mostly consisting of hypothetical proteins with unidentified functions (694), and protein-encoding genes were 254. Tn3 family transposase genes and prophage integrase IntA and IntS were represented in the GIs. **B** Genomic islands in *P. aeruginosa* strains. *P. aeruginosa* ST235 carried GIs comprised of 339 hypothetical proteins and 113 protein-encoding genes comprising of IS1182 family transposase ISCfr1, IS1182 family transposase ISPa7, IS3 family transposase ISPa86, IS3 family transposase ISPosp5, ISL3 family transposase ISStma11, and ISL3 family transposase ISStma11 were present. *P. aeruginosa* ST357 was distinct with 5786 GIs, out of which 3446 were encoded for proteins

*K. pneumoniae* genomes screened against *K. pneumoniae* subsp. *pneumoniae* MGH 78578 predicted common genomic islands mostly consisting of hypothetical proteins with unidentified functions (694), and protein-encoding genes were 254. Tn3 family transposase genes and prophage integrase IntA and IntS were represented in the GIs. GIs were comparable across *K. pneumoniae* strains. AK-624 had GIs comprised of 339 hypothetical proteins and 113 protein-encoding genes. IS1182 family transposase ISCfr1, IS1182 family transposase ISPa7, IS3 family transposase ISPa86, IS3 family transposase ISPosp5, ISL3 family transposase ISStma11, and ISL3 family transposase ISStma11 were present. The distribution of GIs was comparable in AK-625 and AK-631 but significantly varied in AK-628 with 5786 GIs, out of which 3446 were encoded for proteins. A comparative analysis revealed common GIs in *K. pneumoniae* and *P. aeruginosa* strains, respectively (Fig. 3A and B).

### Prophage sequences within the bacterial genome

Sequences of myoviridae and siphoviridae were incorporated into the genome of all the strains.

### Phylogenetic analyses

The phylogenetic relationship was explored based on concatenating 4528 core genes for *K. pneumoniae* strains and 5376 core genes for *P. aeruginosa* strains. The final recombination-free alignment of 21,687 and 95,729 single-nucleotide variants (SNVs) for *K. pneumoniae* and *P. aeruginosa,* respectively, were used to construct a maximum-likelihood phylogenetic tree using Interactive Tree of Life iTOL v 6.7.3 (https://itol.embl.de/). The tree was rooted with NC_009648.1 *K. pneumoniae* subsp. *pneumoniae* MGH 78578 (ATCC® 700,721™) and NZ_CP017149.1 *P. aeruginosa* strain (ATCC® 15,692™) (Figs. 1 and 2).

## Discussion

**Fig. 4:**
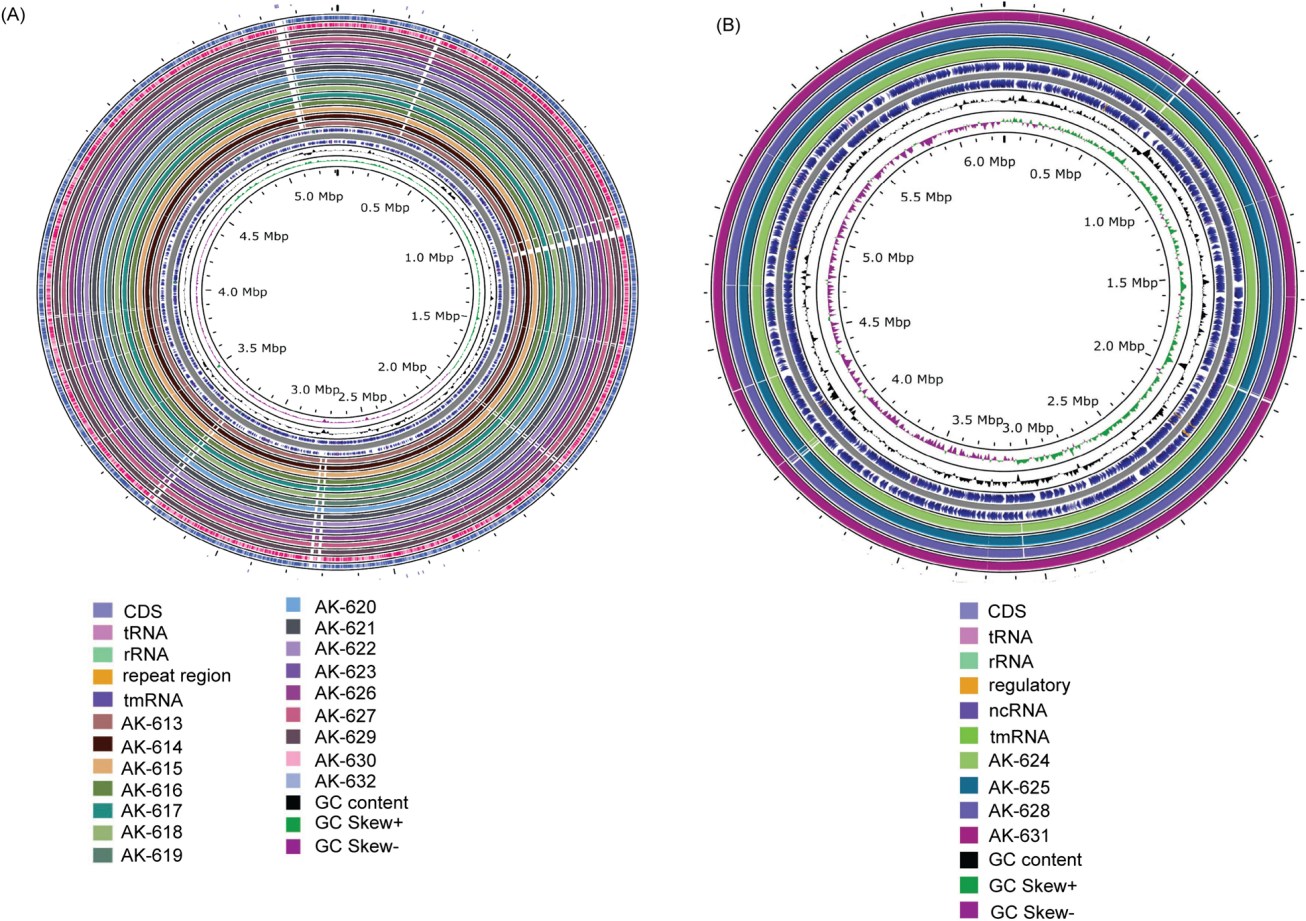
Comparison of whole genome against colistin susceptible strains. **A** Genomes of *K. pneumoniae* strains were compared against colistin susceptible ATCC strain, NC_009648.1 *K. pneumoniae* subsp. pneumoniae MGH 78578 (ATCC® 700,721™). **B** Genomes of *P. aeruginosa* strains were compared against colistin susceptible ATCC strain NZ_CP017149.1 *P. aeruginosa* (ATCC® 15,692™)

The global spread of multi-drug resistant pathogenic bacterial species has become detrimental to the available antimicrobial treatment regimen. The emergence of beta-lactamases such as *bla*_NDM_, extended spectrum beta lactamases (ESBLs) such as *bla*_CTX-M_, *bla*_SHV_, and *bla*_TEM_ etc. has led to the use of last resort antibiotics such as tigecycline, polymyxin E, daptomycin, vancomycin and linezolid [84–86]. However, in the last decade several ARGs against the last-line antibiotics have been reported [87–93]. Colistin is one of the last-resort antibiotics which is administered in cases of MDR Gram-negative bacterial infections. Colistin resistance is a huge challenge that may not be as prevalent as beta-lactamases such as *bla*_NDM_ at present but its emergence and dissemination, especially in hospital settings, is a premonition of impending outbreaks [94]. The inefficiency of standard disk diffusion methods and automated ASTs such as Vitek2 for accurate detection of colistin resistance unified with limited resources and expertise for broth micro-dilution at most hospitals in LMICs such as India demonstrates a low presence of colistin resistance in the country [55]. Next-generation sequencing is an efficient, non-PCR-biased technology that allows for the accurate surveillance of antimicrobial resistance genes and the exploration of their underlying mechanisms. Hospitals are often a hotspot for nosocomial infections and outbreaks. In this study, we analyzed clinical isolates obtained from blood, tracheal aspirate, and pleural fluid of patients admitted to an Indian hospital to assess the prevalence of XDR or PDR bacterial strains exhibiting colistin resistance. While Vitek2 identified only six colistin-resistant isolates, antimicrobial susceptibility testing (AST) using broth micro-dilution revealed 48 isolates as colistin-resistant. Among them, 17/48 (16 *K. pneumoniae* and 1 *P. aeruginosa*) isolates were identified as XDR, and 3/48 (all *P. aeruginosa*) were PDR. Notably, all isolates tested negative for the *mcr*-1 gene. Subsequently, whole-genome sequencing and pan-genomic analyses were conducted to mitigate PCR biases and facilitate a comprehensive exploration of the underlying mechanisms driving colistin resistance. Interestingly, although the strains were isolated from different patients and wards in a hospital (Additional Table 3), all the *K. pneumoniae* strains and *P. aeruginosa* strains displayed remarkable genomic similarities in each ST lineage (Fig. 4A and B). The identical clonal types, capsular type, resistance profile, and plasmids in colistin-resistant *K. pneumoniae* signal a potential XDR *K. pneumoniae* ST147 outbreak in hospital settings (Additional Table 3). The same goes for circulating colistin-resistant *P. aeruginosa* ST235 strains. *K. pneumoniae* ST147 and *P. aeruginosa* ST235 and ST357 are global high-risk clones carrying multiple ARGs [9, 46, 95].

Earlier, *K. pneumoniae* ST147 ColRKp displaying colistin resistance devoid of *mcr* genes has been reported in India. Mutation, truncation, or complete deletion of *mgrB, a* mutation in *pmrB*, *phoQ,* and insertional inactivation have been identified as significant factors inducing colistin resistance [20, 41, 42, 96–98]. Colistin displays bactericidal activity in gram-negative bacteria by binding to the lipid A of LPS. Colistin competitively destabilizes the outer membrane’s surface charge by displacing calcium (Ca2+) and magnesium (Mg2+) divalent cations. Consequently, the three-dimensional structure of LPS is impaired, and permeability is increased, allowing colistin to insert its hydrophobic terminal acyl fat chain. The chromosomally mediated *mgrB* gene is a crucial factor that modulates the surface charge stability to facilitate colistin susceptibility. *mgrB* negatively regulates the functioning of *PhoPQ*, a two-component system involved in LPS modification. The deletion of *mgrB* in all ColRKp might be the major factor driving colistin resistance (Additional Table 5) [16]. Without *mgrB*, *PhoPQ* actively adds the positively charged LAra4N to lipid A, increasing the cationic charge on the surface and prohibiting colistin binding. The phosphoethanolamine transferases add pEtN to lipid A, inhibiting colistin. We detected multiple phosphoethanolamine transferases, namely, *arnT* and *eptB* in *K. pneumoniae* strains and *basS*, *basR*, *arnA*, *cprR,* and *cprS* in *P. aeruginosa* (Figs. 1 and 2). All the strains exhibited amino acid substitutions T246A and R256G in *pmrB* (Additional Table 5). Although earlier studies have also reported these mutations in colistin-resistant strains, they are identified as neutral mutations and believed to have no direct correlation with colistin resistance [99]. As the colistin resistance mechanism is complex, these predictions are not absolutely conclusive, and more investigations, especially at the transcriptional and translational level, are required to understand their relationship with colistin resistance. *lpxM* adds a secondary acyl chain to lipid A, and mutation in *lpxM* can reduce colistin susceptibility in *K. pneumoniae* [18]. The identified mutations in *eptA*, *arnA*, *arnB*, *arnT,* and *kpnF* in *K. pneumoniae* have been reported to increase colistin resistance [100]. The mutations in *arnA*, *arnB*, *arnC*, *arnD*, *arnF*, and *arnT* modify the LPS. The *pmrB* and *parS* mutations in *P. aeruginosa* also increase colistin resistance. His398Arg mutation in *parS* is deleterious, leading to alteration in the functional domain of histidine kinase-like ATPase, which often develops under external colistin stress and ultimately causes colistin resistance [21]. The complete and accurate mechanism of colistin resistance remains unknown. Although no *mcr* gene was detected, the cumulative effect of all these factors reduces colistin susceptibility.

The prevalence of carbapenemases (*bla*_NDM-5_, *bla*_OXA-1_, *bla*_OXA-232_, *bla*_SHV-11_, *bla*_CTX-M-15_ and *LptD*), aminoglycoside resistance genes (*aadA2*, *acrD*, *APH(3″)-Ib*, *APH(6)-Id*, *armA,* and *baeR*), and sulfonamide resistance genes (*sul1*, *sul2*) along with ARGs against other major antibiotic classes in *K. pneumoniae* strains poses a severe threat (Fig. 1A). The porins *ompK35*, *ompK36*, *ompC*, and *ompA* mutations reduce susceptibility towards carbapenem [83]. *ompA* mutation is also involved in colistin resistance. The association of beta-lactamase *bla*_TEM-1_, aminoglycosides *APH(6)-Id* and *APH(3″)-Ib* and *armA*, macrolide resistance genes *msrE* and *mphE*, and sulfonamide resistance gene *sul2* with IS elements in *K. pneumoniae* strains may expedite the horizontal gene transmission. The phosphoethanolamine transferases *cprR* and *cprS* flanked by ISPre2 in *P. aeruginosa* can disseminate colistin resistance-carrying genes horizontally. The resistance of all these clinical strains towards colistin and approximately every other class of antibiotics in clinical settings poses significant challenges due to colistin’s critical status as a last-resort antibiotic. These bacteria’s adaptability and rapid spread of resistance determinants worsen the global healthcare burden [92, 101].

The chi-square test (*p* = 0.007) revealed a significant association between *K. pneumoniae* and *P. aeruginosa* isolates and their prevalence rates. However, an undefined odds ratio was observed due to a lack of representation in the comparison group. Fisher’s Exact Test yielded a *p*-value of 1, indicating no significant association between resistance genes and phenotypic resistance (Additional Table 7B and C). Despite the absence of strains without corresponding resistance genes, further research is needed to fully understand this relationship and develop effective interventions against antimicrobial resistance.

Virulence factors in *K. pneumoniae* and *P. aeruginosa* enhance pathogenicity, attachement to the host cell and colonization, protection against host immune response and biofilm production [48, 53, 102–104]. Globally, *K. pneumoniae* ST147 comprises two major lineages, one with KL10 (wzi420) capsular type and O3 or O3a locus, and another with KL64 (wzi64) capsular type and O2v1 locus [36] Unlike previously reported hospital outbreaks of *K. pneumoniae* ST147 with KL64 capsular typing in Tunisia and Poland, in our study, KL10 (wzi420) was associated [17, 34] (Additional Table 3). KL10 capsulate serotype alongwith V.F.s encoding for Type 1 fimbriae, pili, siderophores and *ompA* increases the *K. pneumoniae* adherence to the host cell and biofilm formation. Biofilm formation protects the underlying bacterial colonies against exogenous stressors and antimicrobial agents [49, 50, 105]. Even though the hypervirulence genes *rmpA/rmpA2* were absent, the virulence factors increasing adherence and penetration were common in all *K. pneumoniae* strains (Additional Table 6).

In *P. aeruginosa*, *exoU* is a critical cytotoxic virulence factor that damages the host cells and is involved in mortality due to *P. aeruginosa* infections [106]. The cascade of alginate proteins in *P. aeruginosa* facilitates biofilm formation, protecting bacteria from antibiotics and immune responses, and hence aiding chronic infections [49]. Further, the presence of Type 4 pilus proteins, type 4 fimbrieae, and twitching motility proteins contributes to its infectivity. These structures play crucial roles in the pathogen’s ability to adhere to and invade host cells, thereby facilitating the initiation and progression of infection. Type 4 pilus proteins enable *P. aeruginosa* to firmly attach to host tissues, promoting colonization and the formation of biofilms. The twitching motility proteins allow *P. aeruginosa* to actively move across surfaces and penetrate deeper into host tissues, facilitating the spread of infection and exacerbating disease severity. This expanded repertoire of virulence factors underscores the formidable pathogenicity of *P. aeruginosa* and its ability to cause various infections, particularly in immunocompromised individuals or those with underlying health conditions. The Type VI Secretion System (T6SS) is a molecular weapon used by *P. aeruginosa* and *K. pneumoniae*, to deliver toxic effector proteins directly into neighboring cells. In *P. aeruginosa*, the T6SS is essential for virulence, interbacterial interactions, and outcompeting other bacteria by delivering toxins into neighboring cells, thereby killing or inhibiting their growth. This competitive advantage is particularly important in environments such as the human body, where *P. aeruginosa* often encounters other microbial species [48, 49, 52, 53]. (Additional Table 6). Biofilm-associated infections in healthcare present challenges due to their resilience, leading to chronicity and increased costs. Biofilms on implanted devices often require removal, while chronic wounds, like diabetic ulcers, face healing delays and complications [107]. In conditions like cystic fibrosis, biofilm-forming *P. aeruginosa* worsen lung function, emphasizing the severity of associated respiratory infections [44, 50]. CRISPR regions in *K. pneumoniae* and *P. aeruginosa* unveiled genomic diversity and assessed immunity against foreign invaders. Presence of CRISPR I enhances survival.

As we explored a limited number of samples from one hospital, it doesn’t give a definite prediction regarding the prevalence of colistin resistance throughout the country. Still, it is a problematic snapshot, with approximately 22% (48/214) colistin-resistant strains. It is in congruence with colistin prevalence in Asia [108]. Our study demonstrates a remarkable similarity among the genomes of colistin-resistant *K. pneumoniae* and *P. aeruginosa* strains, respectively, indicating a potential outbreak from the same source of origin (Figs. 1–4). Due to a lack of real-time surveillance strategies, these outbreaks often go unnoticed in low-middle-income countries. Plasmid-associated ARGs, ARGs flanked by IS elements, multiple virulence factors, genomic islands, and prophage sequences incorporated in bacterial genome ensure a high rate of horizontal gene transfer. Co-resistance of colistin and carbapenem resistance poses a colossal challenge, leaving no alternative antibiotic treatment regimen to the rescue.

### Limitations of the study

The primary limitation of this study lies in its reliance on samples collected solely from a single hospital setting. Consequently, the findings may not fully capture the overall prevalence of colistin resistance and the dissemination of XDR and PDR strains across wider geographic regions or diverse patient populations. While the study coincidentally observed XDR and PDR colistin-resistant strains predominantly among *K. pneumoniae* and *P. aeruginosa* isolates, the exclusive focus on these pathogens inadvertently overlooks other priority pathogens, such as *Acinetobacter baumannii*, which also possess significant resistance capabilities. Consequently, the generalizability of the study’s findings to other healthcare settings or regions may be constrained, necessitating further research encompassing a broader spectrum of pathogens and healthcare contexts for a comprehensive understanding of colistin resistance dissemination.

### Strength of the study

Despite its limitations, this study provides valuable insights into the prevalence and genomic characteristics of colistin-resistant strains, especially among *K. pneumoniae* and *P. aeruginosa* in a hospital setting. Comprehensive genomic analyses, including whole-genome sequencing and pan-genomic analyses, enhance our understanding of colistin resistance dissemination and identify MDR, XDR and PDR strains. Utilizing broth microdilution for antimicrobial susceptibility testing yields more accurate results than Vitek2 systems, improving surveillance and management of antimicrobial resistance. Additionally, despite the absence of the mobile colistin resistance (*mcr*) gene, SNP analysis through whole-genome sequencing reveals novel genetic mechanisms underlying colistin resistance, emphasizing the importance of advanced genomic techniques in combating antimicrobial resistance.

## Conclusions

The global dissemination of MDR pathogenic bacterial species presents a critical challenge to current antimicrobial treatment regimens. The emergence of beta-lactamases such as *bla*_NDM_ and extended-spectrum beta-lactamases (ESBLs) like *bla*_CTX-M_, *bla*_SHV_, and *bla*_TEM_ has necessitated the utilization of last-resort antibiotics such as colistin. However, the rise of resistance against these last-line antibiotics, exemplified by colistin, poses a significant threat, particularly in healthcare settings. The inefficiency of standard detection methods for colistin resistance, coupled with limited resources for comprehensive surveillance, underscores the urgency for improved detection strategies. Through whole-genome sequencing and pan-genomic analyses, we identified a concerning prevalence of colistin-resistant strains, particularly among *K. pneumoniae* and *P. aeruginosa* isolates from a single hospital setting in India. Notably, despite originating from different patients and wards, the genomic similarity among colistin-resistant strains suggests a potential outbreak scenario, emphasizing the importance of real-time surveillance and infection control measures. The detection of extensively drug-resistant (XDR) and pandrug-resistant (PDR) strains among colistin-resistant isolates is concerning. XDR and PDR bacteria resist multiple antibiotic classes, severely limiting treatment options and increasing treatment failure risks. Failure to address XDR and PDR spread could profoundly impact patient care, healthcare resources, and public health. The identification of diverse resistance mechanisms, including mutations in key regulatory genes and plasmid-mediated transfer of resistance determinants, underscores the complexity of combating colistin resistance. Addressing the challenges posed by colistin-resistant MDR, XDR and PDR strains requires a multifaceted approach, encompassing enhanced surveillance, infection control strategies, and the development of novel therapeutic interventions.

### Supplementary Information

**Additional file 1.**

**Additional file 2.**

**Additional file 3.**

**Additional file 4.**

**Additional file 5.**

**Additional file 6.**

**Additional file 7.**

## Data Availability

Data is provided within the manuscript or supplementary information files. The whole genome sequencing data are submitted to the National Center for Biotechnology Information (NCBI) database with BioProject Accession number PRJNA905193.

## Abbreviations

ARG: Antibiotic resistance genes
AMR: Antimicrobial resistance
ST: Sequence type
MLST: Multi locus sequence typing
ColRKp: Colistin resistant *Klebsiella pneumoniae*
