# Supplementary material for: Genome analyses of colistin-resistant high-risk blaNDM-5 producing Klebsiella pneumoniae ST147 and Pseudomonas aeruginosa ST235 and ST357 in clinical settings
Source: BMC Microbiol. 2024 May 20;24:174. doi: 10.1186/s12866-024-03306-4 (PMC11103832; doi:10.1186/s12866-024-03306-4)
Supplement: Supplementary file 1 — Additional file 1. [file 12866_2024_3306_MOESM1_ESM.docx]

**Additional Table 1: MIC of the strains against major classes of antibiotics.**

| **Strains** | **Minimum Inhibitory Concentration (MIC) (µg/ml) for *K. pneumoniae* strains** | | | | | | | | | | | | |
| --- | --- | --- | --- | --- | --- | --- | --- | --- | --- | --- | --- | --- | --- |
|  | CZ | CTR | CPM | CX | AZ | P | I | M | AMI | CPF | L | T | C |
| AK-613 | 2 | 128 | 128 | 128 | >512 | >512 | >512 | 32 | 16 | >512 | 128 | 32 | 128 |
| AK-614 | 1 | 128 | 256 | 128 | 256 | >512 | 256 | 32 | 32 | 256 | 128 | 16 | 128 |
| AK-615 | 1 | 64 | 128 | 64 | 256 | >512 | 256 | 64 | 32 | 256 | 128 | 16 | 64 |
| AK-616 | 1 | 128 | 64 | 64 | 256 | >512 | >512 | 16 | 16 | 256 | 128 | 16 | 64 |
| AK-617 | 1 | 256 | 64 | 64 | 256 | 256 | >512 | 32 | 16 | >512 | 128 | 16 | 64 |
| AK-618 | 2 | 256 | 64 | 128 | 256 | >512 | >512 | 32 | 16 | >512 | 256 | 16 | 128 |
| AK-619 | 2 | 128 | 128 | 128 | >512 | >512 | >512 | 32 | 16 | >512 | 256 | 16 | 64 |
| AK-620 | 2 | 128 | 128 | 256 | >512 | >512 | >512 | 16 | 32 | >512 | 256 | 32 | 128 |
| AK-621 | 2 | 128 | 256 | 128 | 256 | >512 | 256 | 64 | 16 | >512 | 128 | 16 | 128 |
| AK-622 | 1 | 64 | 256 | 256 | 256 | >512 | >512 | 32 | 16 | >512 | 128 | 16 | 128 |
| AK-623 | 4 | 128 | 256 | 256 | 256 | 256 | >512 | 32 | 16 | >512 | 256 | 16 | 128 |
| AK-626 | 2 | 256 | 128 | 128 | >512 | 256 | >512 | 64 | 16 | >512 | 256 | 16 | 128 |
| AK-627 | 2 | 256 | 128 | 128 | 256 | 256 | >512 | 16 | 32 | >512 | 64 | 16 | 128 |
| AK-629 | 4 | 128 | 256 | 256 | >512 | >512 | 256 | 32 | 32 | 256 | 128 | 16 | 64 |
| AK-630 | 2 | 128 | 256 | 128 | >512 | >512 | 256 | 32 | 16 | 256 | 128 | 16 | 64 |
| AK-632 | 4 | 128 | 64 | 256 | >512 | >512 | >512 | 32 | 16 | >512 | 128 | 16 | 64 |
| **Strains** | **Minimum Inhibitory Concentration (MIC) (µg/mL) for *P. aeruginosa* strains** | | | | | | | | | | | | |
|  | G | AMI | I | M | CFZ | CPM | CPF | L | TI | P | AZ | F | C |
| AK-624 | 256 | 128 | 64 | 256 | 128 | 32 | 128 | 128 | >512 | 256 | 256 | 16 | 32 |
| AK-625 | 128 | 64 | 128 | 128 | 128 | 64 | 256 | 64 | 256 | 256 | 128 | 4 | 64 |
| AK-628 | 128 | 128 | 128 | 256 | 256 | 32 | 256 | 64 | >512 | >512 | 128 | 8 | 64 |
| AK-631 | 512 | 64 | >512 | 64 | 64 | 64 | 64 | 128 | 512 | 64 | 128 | 128 | 32 |

All the concentrations are in **µg/mL.** CLSI cut-off for Susceptibility (S) and Resistance (R) against different antibiotics. MIC in *K. pneumoniae*, CZ: Cefazolin (S: ≤2; R: ≥8), CTR: Ceftriaxone, CPM: Cefepime (S: ≤2; R: ≥16), CX: Cefoxitin (S: ≤8; R: ≥32), AZ: Aztreonam (S: ≤4; R: ≥16), P: Piperacillin (S: ≤16; R: ≥128), I: Imipenem (S: ≤1; R: ≥4), M: Meropenem (S: ≤1; R: ≥4), AMI: Amikacin (S: ≤16; R: ≥64), CPF: Ciprofloxacin (S: ≤0.25; R: ≥1), L: Levofloxacin (S: ≤0.5; R: ≥2), T: Tetracycline (S: ≤4; R: ≥16), C: Colistin(R: ≥4 )

MIC in *P. aeruginosa*, G: Gentamicin (S: ≤1; R: ≥4), AMI: Amikacin(S: ≤4; R: ≥16), Imipenem (S: ≤2; R: ≥8), M: Meropenem (S: ≤2; R: ≥8), CFZ: Ceftazidime (S: ≤8; R: ≥32), CPM: Cefepime (S: ≤8; R: ≥32), CPF: Ciprofloxacin (S: ≤0.5; R: ≥2), L: Levofloxacin (S: ≤1; R: ≥4), TI: Ticarcillin-clavulanate (S: ≤16/2; R: ≥128/2), P: Piperacillin (S: ≤16; R: ≥128), AZ: Aztreonam (S: ≤8; R: ≥32), F: Fosfomycin (MIC QC Range for P. aeruginosa ATCC 27853 is 2-8), C: Colistin (R: ≥4 ).
