# Supplementary material for: Genome analyses of colistin-resistant high-risk blaNDM-5 producing Klebsiella pneumoniae ST147 and Pseudomonas aeruginosa ST235 and ST357 in clinical settings
Source: BMC Microbiol. 2024 May 20;24:174. doi: 10.1186/s12866-024-03306-4 (PMC11103832; doi:10.1186/s12866-024-03306-4)
Supplement: Supplementary file 2 — Additional file 2. [file 12866_2024_3306_MOESM2_ESM.docx]

**Additional Table 2: Genomic content in whole genome.**

| **Strain** | **Number of reads (Million)** | **Read Length** | **GC%** | **Genome Coverage** | **Genome size after Primary assembly (bases)** | **Genome size after Secondary assembly**  **(bases)** |
| --- | --- | --- | --- | --- | --- | --- |
| **For *K. pneumoniae* ST147 strains** | | | | | | |
| AK-613 | 11.4 | 150 | 64% | 297.12x | 5771443 | 5363677 |
| AK-614 | 12.4 | 150 | 56% | 325.17x | 5771442 | 5363677 |
| AK-615 | 9.8 | 150 | 56% | 259.36x | 5770749 | 5362493 |
| AK-616 | 1.4 | 150 | 55% | 37.19x | 5763818 | 5362591 |
| AK-617 | 9.2 | 150 | 56% | 185.73x | 12938902 | 4700495 |
| AK-618 | 9 | 150 | 56% | 185.73x | 5771694 | 5363825 |
| AK-619 | 4 | 150 | 55% | 104.01x | 5771652 | 5347394 |
| AK-620 | 19.8 | 150 | 55% | 304.01x | 5771393 | 5363658 |
| AK-621 | 11.2 | 150 | 56% | 296.21x | 5771255 | 5355878 |
| AK-622 | 8.2 | 150 | 56% | 54.64x | 23114044 | 5318120 |
| AK-623 | 7.8 | 150 | 54% | 203.37x | 5811553 | 5351281 |
| AK-626 | 8.2 | 150 | 56% | 214.81x | 5771296 | 5355915 |
| AK-627 | 10.6 | 150 | 56% | 279.06x | 5771783 | 5355991 |
| AK-629 | 15.4 | 150 | 56% | 404.33x | 5771411 | 5363676 |
| AK-630 | 7.6 | 150 | 55% | 198.9x | 5771369 | 5363676 |
| AK-632 | 13.2 | 150 | 55% | 295.21x | 6759363 | 5355703 |
| **For *P. aeruginosa* ST235 (AK-624, AK-625 and AK-628) and ST357 (AK-631) strains** | | | | | | |
| AK-624 | 7.4 | 150 | 64% | 161.62x | 6898984 | 6692442 |
| AK-625 | 10 | 150 | 64% | 219.15x | 6898684 | 6692576 |
| AK-628 | 11 | 150 | 60% | 188.04x | 8897656 | 6692442 |
| AK-631 | 9.8 | 150 | 64% | 224.8x | 6809282 | 6557164 |

GC content was comparable among the isolates in each ST lineage. Primary assembly refers to *de novo* assembly of raw reads, and secondary assembly is obtained by mapping the contigs over a reference genome.
