# Supplementary material for: Genome analyses of colistin-resistant high-risk blaNDM-5 producing Klebsiella pneumoniae ST147 and Pseudomonas aeruginosa ST235 and ST357 in clinical settings
Source: BMC Microbiol. 2024 May 20;24:174. doi: 10.1186/s12866-024-03306-4 (PMC11103832; doi:10.1186/s12866-024-03306-4)
Supplement: Supplementary file 3 — Additional file 3. [file 12866_2024_3306_MOESM3_ESM.docx]

**Additional Table 3**: **Genomic features of colistin-resistant strains**.

| **Strain** | **Sample_type** | **Species** | **ST type** | **K-type** | **wzi** | **O-antigen** | **CRISPR (Spacers)** |
| --- | --- | --- | --- | --- | --- | --- | --- |
| AK-613 | Blood culture | *Klebsiella pneumoniae* | ST147 | KL10 | wzi420 | O3/O3a | General-Class 1 (43) |
| AK-614 | Blood culture | *Klebsiella pneumoniae* | ST147 | KL10 | wzi420 | O3/O3a | General-Class 1 (43) |
| AK-615 | Blood culture | *Klebsiella pneumoniae* | ST147 | KL10 | wzi420 | O3/O3a | General-Class 1 (43) |
| AK-616 | Blood culture | *Klebsiella pneumoniae* | ST147 | KL10 | wzi420 | O3/O3a | General-Class 1 (43) |
| AK-617 | Blood culture | *Klebsiella pneumoniae* | ST147 | KL10 | wzi420 | O3/O3a | General-Class 1 (43) |
| AK-618 | Blood culture | *Klebsiella pneumoniae* | ST147 | KL10 | wzi420 | O3/O3a | General-Class 1 (43) |
| AK-619 | Blood culture | *Klebsiella pneumoniae* | ST147 | KL10 | wzi420 | O3/O3a | General-Class 1 (43) |
| AK-620 | Blood culture | *Klebsiella pneumoniae* | ST147 | KL10 | wzi420 | O3/O3a | General-Class 1 (43) |
| AK-621 | Blood culture | *Klebsiella pneumoniae* | ST147 | KL10 | wzi420 | O3/O3a | General-Class 1 (43) |
| AK-622 | Blood culture | *Klebsiella pneumoniae* | ST147 | KL10 | wzi420 | O3/O3a | General-Class 1 (43) |
| AK-623 | Blood culture | *Klebsiella pneumoniae* | ST147 | KL10 | wzi420 | O3/O3a | General-Class 1 (43) |
| AK-626 | Blood culture | *Klebsiella pneumoniae* | ST147 | KL10 | wzi420 | O3/O3a | General-Class 1 (43) |
| AK-627 | Blood culture | *Klebsiella pneumoniae* | ST147 | KL10 | wzi420 | O3/O3a | General-Class 1 (43) |
| AK-629 | Tracheal aspirate | *Klebsiella pneumoniae* | ST147 | KL10 | wzi420 | O3/O3a | General-Class 1 (43) |
| AK-630 | Blood culture | *Klebsiella pneumoniae* | ST147 | KL10 | wzi420 | O3/O3a | General-Class 1 (43) |
| AK-632 | Blood culture | *Klebsiella pneumoniae* | ST147 | KL10 | wzi420 | O3/O3a | General-Class 1 (43) |
| AK-624 | Blood culture | *Pseudomonas aeruginosa* | ST235 | - | - | O11 | - |
| AK-625 | Pleural fluid | *Pseudomonas aeruginosa* | ST235 | - | - | O11 | - |
| AK-628 | Blood culture | *Pseudomonas aeruginosa* | ST235 | - | - | O11 | - |
| AK-631 | Blood culture | *Pseudomonas aeruginosa* | ST357 | - | - | O11 | General-Class 1 (7),General-Class 1 (9) |

The samples were collected from different sites and wards but had identical capsular and antigen types. The CRISPR sequences were found in all the *K. pneumoniae* strains, but only one *P. aeruginosa* strains, AK-631 carried CRISPR.
