# Supplementary material for: Genome analyses of colistin-resistant high-risk blaNDM-5 producing Klebsiella pneumoniae ST147 and Pseudomonas aeruginosa ST235 and ST357 in clinical settings
Source: BMC Microbiol. 2024 May 20;24:174. doi: 10.1186/s12866-024-03306-4 (PMC11103832; doi:10.1186/s12866-024-03306-4)
Supplement: Supplementary file 4 — Additional file 4. [file 12866_2024_3306_MOESM4_ESM.docx]

**Additional Table 4: Plasmids and IS elements identified through PlasmidFinder v2.1 and Mobile Element Finder v1.0.3**

| **Strains** | **Plasmids** | **IS elements** |
| --- | --- | --- |
| **For *K. pneumoniae* ST147 strains** | | |
| AK-613 | ColKP3,IncFIA(HI1),IncFIB(pKPHS),IncFII,IncR | IS5075,ISEc29,ISKpn1,ISKpn14 |
| AK-614 | ColKP3,IncFIA(HI1),IncFIB(pKPHS1),IncFII,IncR | IS5075,ISEc29,ISKpn1,ISKpn14 |
| AK-615 | ColKP3,IncFIA(HI1),IncFIB(pKPHS1),IncFII,IncR | IS5075,ISEc29,ISKpn1,ISKpn14 |
| AK-616 | ColKP3,IncFIA(HI1),IncFIB(pKPHS1),IncFII,IncR | IS5075,ISEc29,ISKpn1 |
| AK-617 | ColKP3,IncFIA(HI1),IncFIB(pKPHS1),IncFII,IncR | Tn5042,ISPre2,IS5075,ISEc29,ISPst3,ISKpn1,ISKpn14,cn_6612_ISPre2 |
| AK-618 | Col(pHAD28),ColKP3,IncFIA(HI1),IncFIB(pKPHS1),IncFII,IncR | IS5075,ISEc29,ISKpn1,ISKpn14 |
| AK-619 | ColKP3,IncFIA(HI1),IncFIB(pKPHS1),IncFII,IncR | IS5075,ISEc29,ISKpn1,ISKpn14 |
| AK-620 | Col(pHAD28),ColKP3,IncFIA(HI1),IncFIB(pKPHS1),IncFII,IncR | IS5075,ISEc29,ISKpn1,ISKpn14 |
| AK-621 | ColKP3,IncFIA(HI1),IncFIB(pKPHS1),IncFII,IncR | IS5075,ISEc29,ISKpn1,ISKpn14 |
| AK-622 | ColKP3,IncFIA(HI1),IncFIB(pKPHS1),IncFII,IncR | ISPsy25,IS5075,ISEc29 |
| AK-623 | ColKP3,IncFIA(HI1),IncFIB(pKPHS1),IncFII,IncR | ISPsy25,IS5075,ISEc29,ISKpn1,ISKpn14 |
| AK-626 | Col(pHAD28),ColKP3,IncFIA(HI1),IncFIB(pKPHS1),IncFII,IncR | IS5075,ISEc29,ISKpn1,ISKpn14 |
| AK-627 | ColKP3,IncFIA(HI1),IncFIB(pKPHS1),IncFII,IncR | IS5075,ISEc29,ISKpn1,ISKpn14 |
| AK-629 | ColKP3,IncFIA(HI1),IncFIB(pKPHS1),IncFII,IncR | IS5075,ISEc29,ISKpn1,ISKpn14 |
| AK-630 | Col(pHAD28),ColKP3,IncFIA(HI1),IncFIB(pKPHS1),IncFII,IncR | IS5075,ISEc29,ISKpn1,ISKpn14 |
| AK-632 | ColKP3,IncFIA(HI1),IncFIB(pKPHS1),IncFII,IncR | ISAba27,IS5075,IS17,ISEc29,ISKpn1,ISKpn14 |
| **For *P. aeruginosa* ST235 (AK-624, AK-625 and AK-628) and ST357 (AK-631) strains** | | |
| AK-624 | - | ISPa6,ISPa86,IS6100,ISPa7 |
| AK-625 | - | IS-PA-6,IS6100R;IS6100L |
| AK-628 | - | ISPa6,ISEnfa4,ISPa86,IS6100,ISPa7 |
| AK-631 | - | ISPa6,ISPre2,Tn4661,IS26,Tn6196,ISPst3,IS6100 |
