# Supplementary material for: Genome analyses of colistin-resistant high-risk blaNDM-5 producing Klebsiella pneumoniae ST147 and Pseudomonas aeruginosa ST235 and ST357 in clinical settings
Source: BMC Microbiol. 2024 May 20;24:174. doi: 10.1186/s12866-024-03306-4 (PMC11103832; doi:10.1186/s12866-024-03306-4)
Supplement: Supplementary file 5 — Additional file 5. [file 12866_2024_3306_MOESM5_ESM.docx]

**Additional Table 5: Single Nucleotide Polymorphisms (SNPs) involved in colistin resistance.**

1. SNPs in *K. pneumoniae* ST147 strains

| **TYPE** | **AA_POS** | **EFFECT** | **GENE** | **PRODUCT** |
| --- | --- | --- | --- | --- |
| snp | 185/661 | missense_variant c.553A>G p.Thr185Ala | *arnA* | bifunctional UDP-4-amino-4-deoxy-L-arabinose formyltransferase/UDP-glucuronic acid oxidase ArnA |
| complex | 112/379 | missense_variant c.335_336delACinsCA p.Asp112Ala | *arnB* | UDP-4-amino-4-deoxy-L-arabinose aminotransferase |
| snp | 372/551 | missense_variant c.1115G>A p.Arg372Lys | *arnT* | lipid IV(A) 4-amino-4-deoxy-L-arabinosyltransferase |
| snp | 156/551 | missense_variant c.468C>G p.His156Gln | *arnT* | lipid IV(A) 4-amino-4-deoxy-L-arabinosyltransferase |
| snp | 50/546 | missense_variant c.148G>T p.Val50Leu | *eptA* | phosphoethanolamine transferase EptA |
| snp | 135/546 | missense_variant c.403G>C p.Ala135Pro | *eptA* | phosphoethanolamine transferase EptA |
| snp | 138/546 | missense_variant c.412A>G p.Ile138Val | *eptA* | phosphoethanolamine transferase EptA |
| snp | 27/546 | missense_variant c.80G>T p.Cys27Phe | *eptA* | phosphoethanolamine transferase EptA |
| snp | 55/109 | missense_variant c.163G>T p.Ala55Ser | *kpnF* | multidrug efflux SMR transporter subunit KpnF |
| del |  | bidirectional_gene_fusion n.2565059_2565039delGATGCGGAGAGTGGAGTGAAA | *mgrB* | PhoP/PhoQ regulator MgrB |
| snp | 246/365 | missense_variant c.736A>G p.Thr246Ala | *pmrB* | two-component system sensor histidine kinase PmrB |
| snp | 256/365 | missense_variant c.766C>G p.Arg256Gly | *pmrB* | two-component system sensor histidine kinase PmrB |

1. SNPs in *P. aeruginosa* ST235 (AK-624, 625 and 628)

| **TYPE** | **AA_POS** | **EFFECT** | **GENE** | **PRODUCT** |
| --- | --- | --- | --- | --- |
| snp | 376/382 | missense_variant c.1128G>C p.Glu376Asp | *arnB* | UDP-4-amino-4-deoxy-L-arabinose aminotransferase |
| snp | 105/382 | missense_variant c.313C>A p.Arg105Ser | *arnB* | UDP-4-amino-4-deoxy-L-arabinose aminotransferase |
| snp | 302/382 | missense_variant c.905T>C p.Val302Ala | *arnB* | UDP-4-amino-4-deoxy-L-arabinose aminotransferase |
| snp | 327/339 | missense_variant c.980C>T p.Ala327Val | *arnC* | undecaprenyl-phosphate 4-deoxy-4-formamido-L-arabinose transferase |
| snp | 58/295 | missense_variant c.172T>C p.Phe58Leu | *arnD* | 4-deoxy-4-formamido-L-arabinose- phosphoundecaprenol deformylase |
| snp | 272/295 | missense_variant c.815G>A p.Ser272Asn | *arnD* | 4-deoxy-4-formamido-L-arabinose- phosphoundecaprenol deformylase |
| snp | 14/137 | missense_variant c.40G>A p.Val14Met | *arnF* | 4-amino-4-deoxy-L-arabinose-phosphoundecaprenol flippase subunit ArnF |
| snp | 443/549 | missense_variant c.1327A>G p.Thr443Ala | *arnT* | lipid IV(A) 4-amino-4-deoxy-L-arabinosyltransferase |
| snp | 468/549 | missense_variant c.1402G>A p.Val468Met | *arnT* | lipid IV(A) 4-amino-4-deoxy-L-arabinosyltransferase |
| snp | 509/549 | missense_variant c.1525A>G p.Ile509Val | *arnT* | lipid IV(A) 4-amino-4-deoxy-L-arabinosyltransferase |
| snp | 535/549 | missense_variant c.1604G>T p.Arg535Leu | *arnT* | lipid IV(A) 4-amino-4-deoxy-L-arabinosyltransferase |
| snp | 7/549 | missense_variant c.21T>G p.Cys7Trp | *arnT* | lipid IV(A) 4-amino-4-deoxy-L-arabinosyltransferase |
| complex | 151/549 | missense_variant c.451_453delCATinsTAC p.His151Tyr | *arnT* | lipid IV(A) 4-amino-4-deoxy-L-arabinosyltransferase |
| snp | 166/549 | missense_variant c.497C>T p.Thr166Ile | *arnT* | lipid IV(A) 4-amino-4-deoxy-L-arabinosyltransferase |
| snp | 265/549 | missense_variant c.794C>T p.Ala265Val | *arnT* | lipid IV(A) 4-amino-4-deoxy-L-arabinosyltransferase |
| snp | 398/428 | missense_variant c.1193A>G p.His398Arg | *parS* | sensor histidine kinase ParS |
| complex | 85/448 | missense_variant c.254_258delACGACinsTCGAT p.Tyr85Phe | *phoQ* | two-component system sensor histidine kinase PhoQ |
| snp | 71/221 | missense_variant c.212T>G p.Leu71Arg | *pmrA* | two-component system response regulator PrmA |
| snp | 345/477 | missense_variant c.1033T>C p.Tyr345His | *pmrB* | two-component system sensor histidine kinase PmrB |
| snp | 362/477 | missense_variant c.1084G>A p.Gly362Ser | *pmrB* | two-component system sensor histidine kinase PmrB |
| snp | 68/477 | missense_variant c.202G>A p.Gly68Ser | *pmrB* | two-component system sensor histidine kinase PmrB |
| complex | 2/477 | missense_variant c.4_10delTCCCGTGinsCCGCGTA p.SerArgAla2ProArgThr | *pmrB* | two-component system sensor histidine kinase PmrB |

1. SNPs in *P. aeruginosa* ST-357 strain AK-631

| **TYPE** | **AA_POS** | **EFFECT** | **GENE** | **PRODUCT** |
| --- | --- | --- | --- | --- |
| snp | 388/662 | missense_variant c.1162A>G p.Ile388Val | *arnA* | bifunctional UDP-4-amino-4-deoxy-L-arabinose formyltransferase/UDP-glucuronic acid oxidase ArnA |
| snp | 529/662 | missense_variant c.1585G>A p.Val529Ile | *arnA* | bifunctional UDP-4-amino-4-deoxy-L-arabinose formyltransferase/UDP-glucuronic acid oxidase ArnA |
| complex | 311/662 | missense_variant c.933_939delATGCAGTinsGTCCGGC p.CysSer312SerGly | *arnA* | bifunctional UDP-4-amino-4-deoxy-L-arabinose formyltransferase/UDP-glucuronic acid oxidase ArnA |
| snp | 340/382 | missense_variant c.1018C>T p.Arg340Cys | *arnB* | UDP-4-amino-4-deoxy-L-arabinose aminotransferase |
| snp | 375/382 | missense_variant c.1123G>A p.Glu375Lys | *arnB* | UDP-4-amino-4-deoxy-L-arabinose aminotransferase |
| snp | 376/382 | missense_variant c.1128G>C p.Glu376Asp | *arnB* | UDP-4-amino-4-deoxy-L-arabinose aminotransferase |
| snp | 286/382 | missense_variant c.856A>G p.Lys286Glu | *arnB* | UDP-4-amino-4-deoxy-L-arabinose aminotransferase |
| snp | 302/382 | missense_variant c.905T>C p.Val302Ala | *arnB* | UDP-4-amino-4-deoxy-L-arabinose aminotransferase |
| snp | 327/339 | missense_variant c.980C>T p.Ala327Val | *arnC* | undecaprenyl-phosphate 4-deoxy-4-formamido-L-arabinose transferase |
| snp | 272/295 | missense_variant c.815G>A p.Ser272Asn | *arnD* | 4-deoxy-4-formamido-L-arabinose- phosphoundecaprenol deformylase |
| snp | 337/549 | missense_variant c.1010T>A p.Leu337Gln | *arnT* | lipid IV(A) 4-amino-4-deoxy-L-arabinosyltransferase |
| snp | 443/549 | missense_variant c.1327A>G p.Thr443Ala | *arnT* | lipid IV(A) 4-amino-4-deoxy-L-arabinosyltransferase |
| complex | 509/549 | missense_variant c.1525_1527delATTinsGTG p.Ile509Val | *arnT* | lipid IV(A) 4-amino-4-deoxy-L-arabinosyltransferase |
| snp | 7/549 | missense_variant c.21T>G p.Cys7Trp | *arnT* | lipid IV(A) 4-amino-4-deoxy-L-arabinosyltransferase |
| complex | 151/549 | missense_variant c.451_453delCATinsTAC p.His151Tyr | *arnT* | lipid IV(A) 4-amino-4-deoxy-L-arabinosyltransferase |
| snp | 345/477 | missense_variant c.1033T>C p.Tyr345His | *pmrB* | two-component system sensor histidine kinase PmrB |
| snp | 68/477 | missense_variant c.202G>A p.Gly68Ser | *pmrB* | two-component system sensor histidine kinase PmrB |
| complex | 2/477 | missense_variant c.4_10delTCCCGTGinsCCGCGTA p.SerArgAla2ProArgThr | *pmrB* | two-component system sensor histidine kinase PmrB |
| snp | 15/477 | missense_variant c.43G>A p.Val15Ile | *pmrB* | two-component system sensor histidine kinase PmrB |
