# Supplementary material for: Genome analyses of colistin-resistant high-risk blaNDM-5 producing Klebsiella pneumoniae ST147 and Pseudomonas aeruginosa ST235 and ST357 in clinical settings
Source: BMC Microbiol. 2024 May 20;24:174. doi: 10.1186/s12866-024-03306-4 (PMC11103832; doi:10.1186/s12866-024-03306-4)
Supplement: Supplementary file 6 — Additional file 6. [file 12866_2024_3306_MOESM6_ESM.docx]

**Additional Table 6: Virulence factors**

| **Strains** | **Virulence factors** |
| --- | --- |
| **For *K. pneumoniae* ST147 strains** | |
| AK-613 | *entA, entB, entE, entS, fepA, fepB, fepC, fepD, fepG, fimA, fimB, fimE, ompA, yagV/ecpE, yagW/ecpD, yagX/ecpC, yagY/ecpB, yagZ/ecpA, ykgK/ecpR* |
| AK-614 | *entA, entB, entE, fepB, entS, fepD, fepG, fepC, fepA, fimA, fimE, fimB, yagV/ecpE, yagW/ecpD, yagX/ecpC, yagY/ecpB, yagZ/ecpA, ykgK/ecpR, ompA* |
| AK-615 | *entA,entB, entE, fepB, entS, fepD, fepG, fepC, fepA, fimB, fimE, fimA, ykgK/ecpR, yagZ/ecpA, yagY/ecpB, yagX/ecpC, yagW/ecpD, yagV/ecpE, ompA* |
| AK-616 | *ompA, yagV/ecpE, yagW/ecpD, yagX/ecpC, yagY/ecpB, yagZ/ecpA, ykgK/ecpR, fepA, fepC, fepG, fepD, entS, fepB, entE, fimA, fimE, fimB, entA* |
| AK-617 | *mucP, algU, mucD, flgB, flgC, flgG, flgH, flgI, fleQ, fleS, fleR, fliE, fliF, fliG, fliI, fliM, fliN, fliP, fliQ, fliR, flhA, fleN, fliA, motC, pvdS, pvdL, pvdH, mbtH-like, hsiC1/vipB, hsiB1/vipA, pvdA, yagV/ecpE, yagW/ecpD, yagX/ecpC, yagY/ecpB, yagZ/ecpA, ykgK/ecpR, ompA, algB, pilJ, pilH, pilG, pilT, algC, tagR, icmF1/tssM1, dotU1, hsiJ1, hsiB1/vipA, hsiC1/vipB, hcp1, hsiF1, hsiG1, clpV1, vgrG1a, motB, motA, waaA, wag, waaC, waaF, algD, alg8, algG, algI, algA, xcpA/pilD, algW, pvdM, pvdO, pvdF, pvdE, fepA, fepC, fepG, fepD, entS, fepB, entE, entB, entA, fimB, fimE, fimA* |
| AK-618 | *fepA, fepC, fepG, fepD, entS, fepB, entE, entB, entA, fimB, fimE, fimA, yagV/ecpE, yagW/ecpD, yagX/ecpC, yagY/ecpB, yagZ/ecpA, ykgK/ecpR, ompA* |
| AK-619 | *ompA, entA, entB, entE, fepB, entS, fepD, fepG, fepC, fepA, fimB, fimE, fimA, ykgK/ecpR, yagZ/ecpA, yagY/ecpB, yagX/ecpC, yagW/ecpD, yagV/ecpE* |
| AK-620 | *ompA, entA, entB, entE, fepB, entS, fepD, fepG, fepC, fepA, fimA, fimE, fimB, yagV/ecpE, yagW/ecpD, yagX/ecpC, yagY/ecpB, yagZ/ecpA, ykgK/ecpR* |
| AK-621 | *fepA, fepC, fepG, fepD, entS, fepB, entE, entB, entA, fimA, fimE, fimB, ykgK/ecpR, yagZ/ecpA, yagY/ecpB, yagX/ecpC, yagW/ecpD, yagV/ecpE, ompA* |
| AK-622 | *algU,alg8,algI,algA,mbtH-like,pvdH,pvdS,pilH,pilG,dotU1,hsiB1/vipA,hsiC1/vipB,hcp1,hsiG1,clpV1,pvdO,flgC,ykgK/ecpR,yagZ/ecpA,yagY/ecpB,yagX/ecpC,yagW/ecpD,yagV/ecpE,ompA,motC,fliA,fleN,flhA,fliQ,fliP,fliM,fliI,fliG,fleQ,flgI,flgH,flgG,motA,waaF,fepC,fepG,entB,entA* |
| AK-623 | *ompA, entA, entB, entE, fepB, entS, fepD, fepG, fepC, fepA, fimA, fimE, fimB, ykgK/ecpR, yagZ/ecpA, yagY/ecpB, yagX/ecpC, yagW/ecpD, yagV/ecpE* |
| AK-626 | *entA,entB,entE,fepB,entS,fepD,fepG,fepC,fepA,fimA,fimE,fimB,yagV/ecpE,yagW/ecpD,yagX/ecpC,yagY/ecpB,yagZ/ecpA,ykgK/ecpR,ompA* |
| AK-627 | *fepA,fepC,fepG,fepD,entS,fepB,entE,entB,entA,fimB,fimE,fimA,yagV/ecpE,yagW/ecpD,yagX/ecpC,yagY/ecpB,yagZ/ecpA,ykgK/ecpR,ompA* |
| AK-629 | *ompA, entA, entB, fepG, fepC, ykgK/ecpR, yagZ/ecpA, yagY/ecpB, yagX/ecpC, yagW/ecpD, yagV/ecpE* |
| AK-630 | *fepA,fepC,fepG,fepD,entS,fepB,entE,entB,entA,fimB,fimE,fimA,ykgK/ecpR,yagZ/ecpA,yagY/ecpB,yagX/ecpC,yagW/ecpD,yagV/ecpE,ompA* |
| AK-632 | *fepA,fepC,fepG,fepD,entS,fepB,entE,entB,entA,fimB,fimE,fimA,yagV/ecpE,yagW/ecpD,yagX/ecpC,yagY/ecpB,yagZ/ecpA,ykgK/ecpR,ompA* |
| **For *P. aeruginosa* ST235 (AK-624, AK-625 and AK-628) and ST357 (AK-631) strains** | |
| AK-624 | *pilM,pilN,pilO,pilP,pilQ,waaF,waaC,waaG,waaP,waaA,motA,motB,aprA,phzG1,vgrG1b,vgrG1a,clpV1,hsiH1,hsiG1,hsiF1,hsiE1,hcp1,hsiC1/vipB,hsiB1/vipA,hsiA1,fha1,lip1,hsiJ1,dotU1,icmF1/tssM1,tagF/pppB,pppA,ppkA,tagT,tagS,tagR,tagQ,phzH,exoT,phzB1,phzB1,phzM,pvdQ,pvdA,pvdP,pvdM,pvdN,pvdO,pvdF,mbtH-like,pvdH,pvdL,pvdG,pvdS,tse2,exoU,flgB,flgC,flgD,flgE,flgF,flgG,flgH,flgI,flgJ,flgK,fleQ,fleS,fleR,fliE,fliF,fliG,fliH,fliI,fliJ,rhlC,toxA,algB,chpE,chpD,chpC,chpB,chpA,pilK,pilJ,pilI,pilH,pilG,pilU,pilT,motD,motC,fliA,fleN,flhF,flhA,flhB,fliR,fliQ,fliP,fliO,fliN,fliM,fliL,fliK,lasI,pilF,rhlI,rhlB,rhlA,tse3,algC,pilS,pilR,fimT,fimU,pilV,pilW,pilX,pilY1,pilY2,pilE,flgN,flgM,flgA,algZ,algR,algQ,algP/algR3,pvcA,pvcB,pvcC,pvcD,ptxR,plcH,mucD,mucC,mucB,mucA,algU,lasA,tse1,pscL,pscK,pscJ,pscI,pscH,pscG,pscF,pscE,pscD,pscC,pscB,exsD,exsA,exsB,exsE,exsC,popD,popB,pcrH,pcrV,pcrG,pcrR,pcrD,pcr4,pcr3,pcr2,pcr1,popN,pscN,pscO,pscP,pscQ,pscR,pscS,pscT,pscU,pchA,pchB,pchC,pchD,pchR,pchE,pchF,pchG,pchH,pchI,fptA,phzS,mucE,motY,algD,alg8,alg44,algK,algE,algG,algX,algL,algI,algJ,algF,algA,mucP,lasB,algW,pilB,pilC,xcpA/pilD,fimV,xcpQ,xcpP,xcpR,xcpS,xcpT,xcpU,xcpV,xcpW,xcpX,xcpY,xcpZ,phzC1,exoY* |
| AK-625 | *motB,motA,waaA,waaP,waaG,waaC,waaF,pilQ,pilP,pilO,pilN,pilM,aprA,phzG1,vgrG1b,vgrG1a,clpV1,hsiH1,hsiG1,hsiF1,hsiE1,hcp1,hsiC1/vipB,hsiB1/vipA,hsiA1,fha1,lip1,hsiJ1,dotU1,icmF1/tssM1,tagF/pppB,pppA,ppkA,tagT,tagS,tagR,tagQ,phzH,exoT,phzB1,phzB1,phzM,pvdQ,pvdA,pvdP,pvdM,pvdN,pvdO,pvdF,mbtH-like,pvdH,pvdL,pvdG,pvdS,tse2,toxA,rhlC,fliJ,fliI,fliH,fliG,fliF,fliE,fleR,fleS,fleQ,flgK,flgJ,flgI,flgH,flgG,flgF,flgE,flgD,flgC,flgB,exoU,algB,chpE,chpD,chpC,chpB,chpA,pilK,pilJ,pilI,pilH,pilG,pilU,pilT,lasI,fliK,fliL,fliM,fliN,fliO,fliP,fliQ,fliR,flhB,flhA,flhF,fleN,fliA,motC,motD,pilF,tse3,rhlA,rhlB,rhlI,algC,pilS,pilR,fimT,fimU,pilV,pilW,pilX,pilY1,pilY2,pilE,flgA,flgM,flgN,algP/algR3,algQ,algR,algZ,ptxR,pvcD,pvcC,pvcB,pvcA,algU,mucA,mucB,mucC,mucD,plcH,lasA,tse1,pscL,pscK,pscJ,pscI,pscH,pscG,pscF,pscE,pscD,pscC,pscB,exsD,exsA,exsB,exsE,exsC,popD,popB,pcrH,pcrV,pcrG,pcrR,pcrD,pcr4,pcr3,pcr2,pcr1,popN,pscN,pscO,pscP,pscQ,pscR,pscS,pscT,pscU,phzS,fptA,pchI,pchH,pchG,pchF,pchE,pchR,pchD,pchC,pchB,pchA,mucE,lasB,mucP,algA,algF,algJ,algI,algL,algX,algG,algE,algK,alg44,alg8,algD,motY,xcpA/pilD,pilC,pilB,algW,fimV,xcpQ,xcpP,xcpR,xcpS,xcpT,xcpU,xcpV,xcpW,xcpX,xcpY,xcpZ,phzC1,exoY* |
| AK-628 | *motB,motA,waaA,waaP,waaG,waaC,waaF,pilQ,pilP,pilO,pilN,pilM,aprA,exoT,phzH,tagQ,tagR,tagS,tagT,ppkA,pppA,tagF/pppB,icmF1/tssM1,dotU1,hsiJ1,lip1,fha1,hsiA1,hsiB1/vipA,hsiC1/vipB,hcp1,hsiE1,hsiF1,hsiG1,hsiH1,clpV1,vgrG1a,vgrG1b,clpP,phzC1,phzM,pvdS,pvdG,pvdL,pvdH,mbtH-like,pvdF,pvdO,pvdN,pvdM,pvdP,pvdA,pvdQ,tse2,phzG1,exoU,flgB,flgC,flgD,flgE,flgF,flgG,flgH,flgI,flgJ,flgK,fleQ,fleS,fleR,fliE,fliF,fliG,fliH,fliI,fliJ,rhlC,toxA,phzB1,phzB1,pilT,pilU,pilG,pilH,pilI,pilJ,pilK,chpA,chpB,chpC,chpD,chpE,algB,lasI,fliK,fliL,fliM,fliN,fliO,fliP,fliQ,fliR,flhB,flhA,flhF,fleN,fliA,motC,motD,pilF,rhlI,rhlB,rhlA,tse3,algC,plcH,mucD,mucC,mucB,mucA,algU,pilE,pilY2,pilY1,pilX,pilW,pilV,fimU,fimT,pilR,pilS,flgA,flgM,flgN,algP/algR3*,algQ,algR,alg*Z,pvcA,pvcB,pvcC,pvcD,ptxR,pscU,pscT,pscS,pscR,pscQ,pscP,pscO,pscN,popN,pcr1,pcr2,pcr3,pcr4,pcrD,pcrR,pcrG,pcrV,pcrH,popB,popD,exsC,exsE,exsB,exsA,exsD,pscB,pscC,pscD,pscE,pscF,pscG,pscH,pscI,pscJ,pscK,pscL,tse1,lasB,mucP,algA,algF,algJ,algI,algL,algX,algG,algE,algK,alg44,alg8,algD,motY,lasA,algW,pilB,pilC,xcpA/pilD,pchA,pchB,pchC,pchD,pchR,pchE,pchF,pchG,pchH,pchI,fptA,phzS,mucE,xcpZ,xcpY,xcpX,xcpW,xcpV,xcpU,xcpT,xcpS,xcpR,xcpP,xcpQ,fimV,exoY* |
| AK-631 | *pilF,lasB,mucP,algA,algF,algJ,algI,algL,algX,algG,algE,algK,alg44,alg8,algD,motY,phzB1,phzB1,tse3,rhlA,rhlB,rhlI,flgN,flgM,flgA,exoY,mucE,phzM,algB,pilR,pilS,algZ,algR,algQ,algP/algR3,pvdF,pvdO,pvdN,pvdM,pvdP,pvdA,pvdQ,motD,motC,fliA,fleN,flhF,flhA,flhB,fliR,fliQ,fliP,fliO,fliN,fliM,fliL,fliK,lasI,aprA,toxA,rhlC,fliJ,fliI,fliH,fliG,fliF,fliE,fleR,fleS,fleQ,flgK,flgJ,flgI,flgH,flgG,flgF,flgE,flgD,flgC,flgB,exoU,algC,ptxR,pvcD,pvcC,pvcB,pvcA,phzH,exoT,mbtH-like,pvdH,pvdL,pvdG,pvdS,phzS,fptA,pchI,pchH,pchG,pchF,pchE,pchR,pchD,pchC,pchB,pchA,motB,motA,waaA,waaP,waaG,waaC,waaF,pilQ,pilP,pilO,pilN,pilM,tse2,tagQ,tagR,tagS,tagT,ppkA,pppA,tagF/pppB,icmF1/tssM1,dotU1,hsiJ1,lip1,fha1,hsiA1,hsiB1/vipA,hsiC1/vipB,hcp1,hsiE1,hsiF1,hsiG1,hsiH1,clpV1,vgrG1a,xcpZ,xcpY,xcpX,xcpW,xcpV,xcpU,xcpT,xcpS,xcpR,xcpP,xcpQ,fimV,pilT,pilU,pilG,pilH,pilI,pilJ,pilK,chpA,chpB,chpC,chpD,chpE,plcH,mucD,mucC,mucB,mucA,algU,phzF1,phzG1,lasA,tse1,pscL,pscK,pscJ,pscI,pscH,pscG,pscF,pscE,pscD,pscC,pscB,exsD,exsA,exsB,exsE,exsC,popD,popB,pcrH,pcrV,pcrG,pcrR,pcrD,pcr4,pcr3,pcr2,pcr1,popN,pscN,pscO,pscP,pscQ,pscR,pscS,pscT,pscU,phzC1,phzD1,algW,pilB,xcpA/pilD* |
