# Supplementary material for: Genome analyses of colistin-resistant high-risk blaNDM-5 producing Klebsiella pneumoniae ST147 and Pseudomonas aeruginosa ST235 and ST357 in clinical settings
Source: BMC Microbiol. 2024 May 20;24:174. doi: 10.1186/s12866-024-03306-4 (PMC11103832; doi:10.1186/s12866-024-03306-4)
Supplement: Supplementary file 7 — Additional file 7. [file 12866_2024_3306_MOESM7_ESM.docx]

Additional Table 7 A: The occurrence of multi-drug resistance (MDR), extremely drug resistance (XDR) and pan-drug resistance (PDR), and multiple antibiotic resistance (MAR) index among the recovered isolates.

| Bacterial pathogen | No. of strains (%) | Type of resistance | MAR index | Phenotypic multidrug resistance | Antibiotic and corresponding Antibiotic resistance Genes |
| --- | --- | --- | --- | --- | --- |
| *K. pneumoniae* | 16 | XDR | 11/13=0.846 | **Cefepime**: Ceftriaxone, Cefepime, Cefoxitin, Aztreonam, **Penicillin:** Piperacillin, **Carbapenem**:  Imipenem, Meropenem, **Fluoroquinolones:** Ciprofloxacin, Levofloxacin, **Tetracycline**, **Polymyxin:** Colistin | **Aminocoumarin**: *mdtC*;  **Aminoglycoside***: APH(6)-Id, APH(3'')-Ib, armA, acrD, aadA2, baeR, smeA, smeD, smeE, AAC(6')-Ib-cr6,*  *smeR*;  **Carbapenem**: *NDM-5, SHV-11, OXA-232, OXA-1, LptD*;  Cephalosporin:  *CTX-M-15*;  **Diaminopyrimidine**: *dfrA12;* **disinfecting agents and antiseptics:** *qacEdelta1*;  **Fluoroquinolone**: *Escherichia coli* *parC* conferring resistance to fluoroquinolones, *emrR,* *QnrB1,* *acrB*, *oqxB,* *oqxA,* *marA*;  **Glycopeptide**: *BRP(MBL)*;  **Macrolide:** *mphE, EreA2, mphA, Klebsiella pneumoniae KpnF, Klebsiella pneumoniae KpnE, Klebsiella pneumoniae KpnH, Klebsiella pneumoniae KpnG, CRP, ErmB, msrE*;  **Monobactam:** *Klebsiella pneumoniae OmpK37*  *TEM-1*; **Nitroimidazole:** *msbA*; **Peptide:** *eptB, ArnT, OmpA*;  **Phenicol:**  *cmlA5*;  **Phosphonic acid:** *FosA6*,  *Escherichia coli UhpT* with mutation conferring resistance to fosfomycin; **Rifamycin:** *arr-2*;  **Sulfonamide**: *sul2, sul1*;  **Tetracycline:** *tet(39*). |
| *P. aeruginosa* | 3  1 | PDR  XDR | 13/13=1  12/13=0.923 | **Aminoglycoside:**  Gentamicin, Amikacin, **Carbapenem:**  Imipenem, Meropenem, **Cephalosporin:**  Ceftazidime, Cefepime, **Fluoroquinolone**:  Ciprofloxacin, Levofloxacin, **Penicillin/Beta-lactamase inhibitor combination:** Ticarcillin-clavulanate, **Penicillin:** Piperacillin, **Monobactam**: Aztreonam, **Phosphonic acid:** Fosfomycin,  **Polymyxin:** Colistin | **Aminoglycoside**: *Pseudomonas aeruginosa emrE, APH(3')-IIb, ANT(2'')-Ia, AAC(6')-Il, ANT(3'')-IIa,* *APH(3')-VI, ANT(4')-IIb, aadA6;* **Bicyclomycin-like antibiotic:** bcr-1; **Carbapenem:** NDM-1, VIM-2, OXA-488, OXA-846, OXA-10 ; **Disinfecting agents and antiseptics:** *OpmH*, *TriC*, *TriB*, *TriA*, *qacEdelta1*; **Fluoroquinolone:** *qacEdelta1, Pseudomonas aeruginosa gyrA conferring resistance to fluoroquinolones, PmpM, YajC,* *Pseudomonas aeruginosa soxR,* *MexS,MexT,MexE,MexF,OprN,rsmA,MexG,MexH,MexI,OpmD*;**Macrolide:** *opmE,mexQ,mexP*, *MexZ*, *mexY*, *OprJ*, *MexD*, *MexC*, *Type B, NfxB*, *OprM*, *Pseudomonas aeruginosa CpxR*, *ParR*, *ParS*,*MexB*,*MexA,MexR,ArmR*,*nalC,MexV,MexW*,*OpmB*,*MuxC*,*MuxB*,*MuxA*,*MexK*,*MexJ*,*MexL,* *mphE,msrE* ;  **Monobactam:** PDC-35; **Peptide**: *basS*, *basR,arnA*,*cprS*,*cprR*; **Phenicol:** *mexN*,*mexM*,*Pseudomonas aeruginosa catB7, cmlA5*; **Phosphonic acid**: *FosA*; **Sulfonamide:** *sul1*;  **Glycopeptide:** *BRP(MBL)*;  **Diaminopyrimidine**: *dfrG*  *dfrB2*;  **Lincosamide:** lnuA; **Tetracycline**: tet(A); **Rifamycin**: *arr-3* |

Additional Table 7B: The correlation between phenotypic and genotypic multi-drug resistance in *K. pneumoniae* strains.

| Phenotypic Resistance | Resistance Genes | Strains Resistant & Carrying Gene | Strains Resistant & Not Carrying Gene | Odds Ratio |
| --- | --- | --- | --- | --- |
| Cephalosporin | *CTX-M-15* | 16 | 0 | NA |
| Carbapenem | *NDM-5, SHV-11, OXA-232, OXA-1, LptD* | 16 | 0 | NA |
| Fluoroquinolone | *Escherichia coli parC conferring resistance to fluoroquinolones, emrR, QnrB1, acrB, oqxB, oqxA, marA* | 16 | 0 | NA |
| Phenicol | *cmlA5* | 16 | 0 | NA |
| Tetracycline | *tet(39)* | 15 | 1 | 15 |

The correlation between phenotypic and genotypic multi-drug resistance in *P. aeruginosa* strains.

| Phenotypic Resistance | Resistance Genes | Strains Resistant & Carrying Gene | Strains Resistant & Not Carrying Gene | Odds Ratio |
| --- | --- | --- | --- | --- |
| Aminoglycoside | *Pseudomonas aeruginosa emrE, APH(3')-IIb, ANT(2'')-Ia, AAC(6')-Il, ANT(3'')-IIa, APH(3')-VI, ANT(4')-IIb, aadA6* | 4 | 0 | NA |
| Carbapenem | *NDM-1, VIM-2, OXA-488, OXA-846, OXA-10* | 4 | 0 | NA |
| Fluoroquinolone | *qacEdelta1, Pseudomonas aeruginosa gyrA conferring resistance to fluoroquinolones, PmpM, YajC, Pseudomonas aeruginosa soxR, MexS, MexT, MexE, MexF, OprN, rsmA, MexG, MexH, MexI, OpmD, opmE, mexQ, mexP, MexZ, mexY, OprJ, MexD, MexC, Type B, NfxB, OprM, Pseudomonas aeruginosa CpxR, ParR, ParS, MexB, MexA, MexR, ArmR, nalC, MexV, MexW, OpmB, MuxC, MuxB, MuxA, MexK, MexJ, MexL, mphE, msrE* | 4 | 0 | NA |
| Monobactam | *PDC-35* | 3 | 1 | 3 |
| Phosphonic acid | *FosA* | 4 | 0 | NA |
